# Screening of Lesser-Known Salted–Dried Fish Species for Fatty Acids, Tocols, and Squalene

**DOI:** 10.3390/foods12051083

**Published:** 2023-03-03

**Authors:** Svetlana Lyashenko, Tarik Chileh-Chelh, Miguel Ángel Rincón-Cervera, Svetlana P. Lyashenko, Zalina Ishenko, Oleg Denisenko, Valentina Karpenko, Irene Torres-García, José Luis Guil-Guerrero

**Affiliations:** 1 Food Technology Division, ceiA3, CIAMBITAL, University of Almería, 04120 Almería, Spain; 2 Institute of Nutrition and Food Technology, University of Chile, 7830490 Macul, Chile; 3 Pyatigorsk Medical and Pharmaceutical Institute, Branch of Volgograd State Medical University, 357500 Pyatigorsk, Russia

**Keywords:** salted–dried fish, omega-3 fatty acids, DHA, EPA, tocopherols, squalene, nutritional quality indices

## Abstract

The fillets and roes of 29 species of dry-salted fishes consumed in Eurasian countries were analyzed for fatty acids (FAs), tocols, and squalene, looking for derived health benefits. FAs were analyzed by GC-FID, and tocols and squalene were analyzed by HPLC-DAD. With some exceptions, docosahexaenoic (DHA, 22:6*n*-3), eicosapentaenoic (EPA, 20:5*n*-3), and arachidonic (ARA, 20:4*n*-6) acids were the prominent polyunsaturated fatty acids (PUFAs). The fillets of *Scardinius erythrophthalmus* reached the highest amounts of total FAs, ARA, and DHA (23.1, 1.82, and 2.49 mg/100 g). The fillets of *Seriola quinqueradiata* showed the highest percentages of DHA (34.4% of total FAs). Nutritional quality indices for fish lipids were favorable in all samples, especially the *n*-6/*n*-3 PUFA ratio, which was below 1 in most cases. α-Tocopherol was found in all fillets and roes, especially in Cyprinidae and Pleuronectidae species, and the highest value was found in the roes of *Abramis brama* (5.43 mg/100 g). Most samples contained tocotrienols at trace levels. The fillets of *Clupeonella cultriventris* contained the highest amounts of squalene (1.83 mg/100 g). Overall, dry-salted fish stand out due to their high concentrations of ARA, EPA, and DHA, as well as for α-tocopherol concentrations in roes.

## 1. Introduction

Fish are among the most perishable food items, and several methods have been implemented worldwide for preserving them. Fish drying is a simple and inexpensive preservation method usually employed in many European and Asian countries. Drying implies the removal of water from the fish body through evaporation by exposure to the sun and air flow, which gives characteristic color, texture, and flavor to dried fishes [1]. The consumer preference towards dried fish products is not only because of their traditionally pleasant taste and flavor, but also due to their high amounts of *n*-3 (omega-3) polyunsaturated fatty acids (*n*-3 PUFAs), which are perceived by the population as health-promoting nutrients. *n*-3 PUFAs improve health by decreasing the risk of cardiovascular diseases (CVDs); reducing serum triacylglycerol levels, blood pressure, and insulin resistance; modulating the glucose metabolism and inflammatory processes; and developing a neuroprotective role [2]. 

Many fish species are known to be excellent dietary sources of different *n*-3 and *n*-6 PUFAs, particularly arachidonic acid (ARA, 20:4*n*-6), eicosapentaenoic acid (EPA, 20:5*n*-3), and docosahexaenoic acid (DHA, 22:6*n*-3) [3]. ARA plays an essential role in the human body since it is the precursor of eicosanoids that modulate inflammation and are involved in platelet aggregation and blood clotting. EPA and DHA are involved in many physiological functions such as the fluidity of cell membranes and gene expression. These PUFAs decrease the risk of cardiovascular diseases (CVDs) and neurological disorders and ameliorate the harmful effects of fatty liver and oxidative stress. Furthermore, both of these *n*-3 PUFAs are the metabolic precursors of lipid mediators having anti-inflammatory properties [4]. Although EPA and DHA can be synthesized in the human body from their metabolic precursor α-linolenic acid (ALA, 18:3*n*-3), the conversion rate is very low because of the limited activity of the Δ6-desaturase enzyme, which is involved in the metabolic pathway from ALA to long-chain *n*-3 PUFAs (LCPUFAs). Thus, both EPA and DHA should be included in the diet, and marine foods are the most important sources [5]. The European Food Safety Authority (EFSA) gave recommendations for *n*-3 LCPUFAs in 2010 [6]. For adults, an adequate intake of EPA + DHA was established at 250 mg/day; for infants and young children, it was set at 100 mg/day for DHA; and for pregnancy and lactation, it was 100–200 mg of DHA. However, higher doses of EPA and DHA (1–2 g/day) may be needed to achieve therapeutic effects in secondary prevention strategies for CVD [7].

Dry-salted fishes are consumed as snacks worldwide; however, little information is available regarding the nutritional quality of these products, particularly concerning tocol (Tc) and squalene (Sq) contents. The interest in characterizing Tc content in foods has increased in the last decades, probably due to the awareness of their health impact. Tocols, which comprise tocopherols (T’s) and tocotrienols (T_3_’s), are essential compounds for human nutrition because of their vitamin E and antioxidant bioactivities [8]. Tocols prevent lipid peroxidation by acting as reactive oxygen species scavengers [9], possess antitumor and anti-inflammatory activities, and prevent CVD and diabetes [10]. On the other hand, Sq displays antioxidant and anti-inflammatory properties, and its intake could be useful for the treatment and prevention of CVD [11]. 

The aim of this work was to assess the FA profiles, lipid quality indices, and Tc and Sq contents of several dry-salted fishes and roes worldwide consumed. Improving knowledge on this subject will contribute to expanding the limited information available on the nutritional quality of lipids contained in such food products.

## 2. Material and Methods

### 2.1. Solvents and Reagents

Unless otherwise stated, solvents and reagents were purchased from Sigma-Aldrich (Barcelona, Spain).

### 2.2. Samples

Data regarding samples analyzed in the current study are shown in Table 1. Twenty-eight samples of salted–dried fish and seven samples of roes were selected, resulting in twenty-nine commercially important fish species. The production process of the salted fish analyzed in this work is regulated in the Russian Federation by GOST R 51574-2000 standards. The process can be found at https://docs.cntd.ru/document/1200064665. In this process, the mass fraction of table salt in fish meat fits within the 3–6% range.

Each species was purchased in five different local markets in the city of Essentuki (Russian Federation), all of them coming from the same industrial process. Individual samples were analyzed separately in triplicate, and average values ±SD calculated for five samples of each species are detailed in tables.

### 2.3. Moisture Content

This procedure is described in Supplementary File S1.

### 2.4. Fatty Acid Analyses

The FA profiles were obtained after direct derivatization of the FAs contained in samples to FA methyl esters (FAMEs), as described in a recent paper by our research group [12]. FAME analyses were performed using a Focus GC (Thermo Electron, Cambridge, UK) equipped with a flame ionization detector (FID) and an Omegawax 250 capillary column. This methodology is fully described in Supplementary File S1.

### 2.5. Nutritional Quality Indices of Lipids

Six indicators based on FA composition were estimated as nutritional quality indices of sampled foods: *n*-6/*n*-3 ratio, PUFA/saturated FA (SFA) ratio, atherogenic index (AI), thrombogenic index (TI), hypocholesterolemic/hypercholesterolemic FA ratio (HH), and fish lipid quality (FLQ). AI, TI, HH, and FLQ were calculated according to [13]. To calculate the nutritional indices, the FA concentrations were expressed as mg/100 g.

AI = [12:0 + (4 × 14:0) + 16:0]/Σ Unsaturated FA TI = (14:0 + 16:0 + 18:0)/[(0.5 × Σ MUFA) + (0. 5× Σ n−6 PUFA) + (3 × Σ n−3 PUFA) + (n−3/n−6)] HH = (*cis*−18:1+ Σ PUFA)/(12:0 + 14:0 + 16:0) FLQ =100 × (22:6 n−3 + 20:5 n−3)/ΣFA

### 2.6. Extraction of Tocols and Squalene

This was carried out as described in a previous paper from our research group [14], and is fully described in Supplementary File S1.

### 2.7. Analysis of Tocols and Squalene

T and T_3_ homologs were determined using an RP-HPLC instrument (Agilent 1100 series, Palo Alto, CA, USA) equipped with a diode array detector (DAD) and a ProntoSIL C30 column (4.6 × 250 mm, 3 μm; Bischoff Chromatography, Leonberg, Germany) according to [14]. Sq was determined by RP-HPLC/DAD using a Luna C18 column (250 × 4.6 mm, 5 μm; Phenomenex) at a fixed temperature of 30 °C. This methodology is fully described in Supplementary File S1.

Tocopherol and tocotrienol contents were used for vitamin E activity (VEA) calculation [15], according to the following equation:

VEA = α-T + (β-T*0.5) + (γ-T*0.1) + (δ-T*0.03) + (α-T_3_*0.3) + (β-T_3_*0.05) + (γ-T_3_*0.01)

where different T’s and T_3_’s corresponded to the different tocopherol and tocotrienol contents, respectively, expressed as µg/g. The results were expressed as α-T equivalents (µg/g).

### 2.8. Statistical Analysis

Mean values of three samples analyzed in triplicate are reported as mean value ± SD in tables. Normally distributed data was assessed using a Shapiro–Wilk test, and variance homogeneity was verified using Levene’s test. All data were analyzed using one-way ANOVA (Statgraphics Centurion XVI.I, Warrenton, VA, USA). Significant differences among mean values were checked through Duncan’s test (*p* < 0.05).

## 3. Results

### 3.1. Moisture Content

The moisture content of samples is detailed in Table 2. In fish fillets, it ranged from 12.2 (*Aspius aspius*) to 20.3 g/100 g (*Pleuronectes quadrituberculatus*). In roe samples, the range was between 20.4 (*Scardinius erythrophthalmus*) and 25.6 g/100 g (*Osmerus mordax*).

### 3.2. Total Fatty Acid Content

The total FAs of sampled fishes and roes are summarized in Table 2. Among fishes, the total FA amount ranged between 1.7 in *Ballerus sapa* and 23.1 g/100 g in *S. erythrophthalmus.* Among roe samples, the lowest FA contents were detected in *Abramis brama* and *O. mordax* (2.9 g/100 g), whereas the roes from *Cyrprinus carpio* and *S. erythrophthalmus* were at the top of the range (12.8 g/100 g). Overall, the FA content was generally higher in fish than in roes for the same species: the ratio of FA content between fish and roes from *A. brama, Rutilus caspicus, O. mordax*, and *P. quadrituberculatus* ranged between 1.40 and 1.50, whereas for *S. erythrophthalmus*, this value was 1.80. The only exception to this trend was *C. carpio*, which showed a lower FA content in fish than in roes (ratio 0.62). *S. erythrophthalmus* was the species with the highest FA content in fillets and roes. 

### 3.3. Fatty Acid Profiles

Data on FA content are shown in Table 2 (FA profiles) and Supplementary Table S1 (FA groups). SFAs ranged between 20.6% (*Gadus morhua*) and 39.6% (*Hypophthalmichthys molitrix*) in the fillet samples and between 26.4% (*P. quadrituberculatus*) and 36.1% (*S. erythrophthalmus*) in the roe samples. SFAs reached higher percentages than monounsaturated FAs (MUFAs) and PUFAs in the roes of *C. carpio*, *H. molitrix*, *Mullus barbatus*, and *Laemonema longipes*. Palmitic acid (PA, 16:0) was the main SFA in almost all cases, and only the fillets of *C. carpio* showed similar proportions of PA and stearic acid (SA, 18:0). 

MUFAs ranged between 12.3 (*Seriola quinqueradiata*) and 49.3% (*Clupeonella cultriventris*). This was the most abundant FA group in *C. cultriventris*, *Alosa kessleri*, *Rutilus heckelii*, *Pelecus cultratus*, *A. aspius*, *Blice bjoerkna*, *Parasilurus asotus*, *P. quadrituberculatus*, and *Alburnus mento*, and in the roes of *O. mordax*. Oleic acid (OA, 18:1*n*-9) was the main MUFA in all samples, except in *P. quadrituberculatus*, which contained palmitoleic acid (POA, 16:1*n*-7) as the main MUFA. 

PUFAs ranged between 13.1 (*A. aspius*) and 49.7% (*S. quinqueradiata*) and were the most abundant FA group in both fillets and roes. In most cases, *n*-3 PUFA percentages were higher than *n*-6 ones. With some exceptions, DHA, EPA, and ARA were the most abundant PUFAs. The fillets of *S. quinqueradiata, G. chalcogrammus*, and *Perca fluviatilis* showed the highest amounts of DHA, with 34.4, 31.5, and 30.6% of total FAs, respectively, while in roes, DHA ranged from 10.5 (*O. mordax*) to 22.6% (*R. caspicus*) of total FAs. The highest percentages of EPA were found in the fillets of *Pleuronectes* *quadrituberculatus* and *G. morhua* and in the roes of *P. quadrituberculatus* (21.3, 17.9, and 18.8% of total FAs, respectively). Docosapentaenoic acid (DPA, 22:5*n*-3) was found in proportions ≥5.0% in three samples of fillets (*C. carpio*, *S. quinqueradiata*, and *Perca fluviatilis*) and three samples of roes (*L. longipes*, *P. quadrituberculatus*, and *C. carpio*). The fillets of *A. aspius* and *Barbus tauricus* showed the lowest percentages of EPA + DHA (8.8 and 8.9%), whereas *S. quinqueradiata* (39.4), *Gadus chalcogrammus* (39.8), and *G. morhua* (41.0%) showed the highest EPA + DHA proportions. Roe samples showed intermediate values for EPA + DHA percentages (from 16.6 in *L. longipes* to 37.3% in *P. quadrituberculatus*). DHA percentages were in most cases higher than EPA percentages.

### 3.4. Nutritional Quality Indices of Lipids

Six nutritional indices were calculated (Supplementary Table S2). The PUFA/SFA ratio ranged between 0.36 (*A. aspius*) and 2.15 (*G. morhua*). In roe samples, this ratio was between 0.84 (*L. longipes*) and 1.74 (*P. quadrituberculatus*). The *n*-6/*n*-3 PUFA ratio was below 1, except in *B. tauricus* fillets (1.52). AI and TI were below 1 in all cases, ranging from 0.24 (*B. tauricus*) to 0.73 (*H. molitrix*) and from 0.15 (*P. quadrituberculatus*) to 0.57 (*P. cultratus*) in fillets, respectively. On the other hand, *A. aspius* and *B. tauricus* fillets showed the lowest (1.80) and highest (4.85) HH values, respectively, while FLQ was between 9.70 (*A. aspius* fillets) and 42.10 (*P. quadrituberculatus* roes). Other samples having good FLQ values were the fillets of *G. morhua* (41.88), *S. quinqueradiata* (40.41), and *G. chalcogrammus* (39.84).

### 3.5. Tocol and Squalene Contents

T and Sq amounts are detailed in Table 3. Among T isoforms, α-T was found in all fillets and roes. The samples having the highest α-T values were the roes of *A. brama* and *P. quadrituberculatus* (5.43 and 6.23 mg/100 g). The lowest amounts of α-T were found in the fillets of *M. barbatus* and *O. mordax* (0.16 mg/100 g) and in the roes of *L. longipes* (0.89 mg/100 g). γ-T was found only in small amounts in the fillets of *B. tauricus*, *Ballerus ballerus*, and *P. quadrituberculatus* (0.18, 0.04, and 0.04 mg/100 g), as well as in the roes of *A. brama* (0.19 mg/100 g). β- and δ-T were below the detection limit in all samples. 

Regarding T_3_’s, β- and γ-T_3_ were found in most samples at trace levels, that is, detected but below the LOQ. It was only possible to quantify α-T_3_ in the roes of *A. brama* and *P. quadrituberculatus* (0.09 and 0.08 mg/100 g), and γ-T_3_ was quantified in the fillets of *B. tauricus* and *S. erythrophthalmus* (0.05 and 0.06 mg/100 g).

The VEA, expressed as α-T equivalents, ranged from 1.6 in the fillets of *M. barbatus* and *O. mordax* to 6.26 mg/100 g in the roes of *P. quadrituberculatus*. The VEA range for fillets (from 0.16 in *M. barbatus* to 3.15 mg/100 g in *P. quadrituberculatus*) was lower than that for roes (from 0.89 in *L. longipes* to 6.26 mg/100 g) due to the low α-T content of the former, which is the most active vitamer. 

Sq was found in variable amounts in 29 out of 35 analyzed samples, and the highest concentrations were detected in the fillets of *C. cultriventris* and *S. quinqueradiata* (1.83 and 1.54 mg/100 g). All roes contained this compound, and the range was between 0.05 (*S. erythrophthalmus*) and 0.70 mg/100 g (*R. caspicus*); for fillets, Sq ranged from undetectable levels in eight samples to 1.83 mg/100 g in *C. cultriventris*.

## 4. Discussion

### 4.1. Total Fatty Acid Content

The highest amounts of total FAs were found in the fillets of *Clupeonella cultriventris* (19.7) and *S. erythrophthalmus* (23.1 g/100 g), both of marine origin (Table 1), while most of the analyzed fish of fluvial origin had low total FA values. The total FA amounts of the former agree with those shown by salted mackerel, which is the name for over 30 species of pelagic or midwater-dwelling fish belonging to the Scombridae family. For this type of marine/fatty fish, the USDA Nutrient Database (FDC ID: 168149) indicates 21.7 g of fat per 100 g of fillet [16], which is in good agreement with the best fatty fishes reported here.

### 4.2. Fatty Acid Composition

Concerning *n*-3 PUFAs, DHA was especially abundant in the families Carangidae (~25–34% of total FAs), Gadidae (~23–32%), Osmeridae (~23–38%), and Percidae (~31%). EPA stands out in roes of the Osmeridae and Pleuronectidae species (16.4 and 18.8%) and in fillets of the Pleuronectidae (21.3%) and Gadidae (8–18%) species. As for *n*-6 PUFAs, ARA reached the maximum percentages in the roes of Cyprinidae and Moridae species (~4–7%) and in fillets of Mulllidae (4.8%). Considering *n*-9 MUFAs, OA was the predominant FA of Clupeidae species (~30–31%). As for FA groups (Supplementary Table S1), *n*-3 PUFAs were the outstanding group in the families Gadidade (~42–44%), Percidae (42.5%), and Carangidae (37–47%), while the roes of Pleuronectidae also showed high percentages (43%). The remaining FA groups did not show a clear tendency among families and analyzed organs. This distribution of the various FAs in fish species was as expected. For example, fish from cold sea families accumulate PUFAs to maintain the fluidity of cell membranes, as occurs with the species of Gadidae, Osmeridae, and Percidae analyzed here. Conversely, OA-rich Cupleidae species occur in more temperate waters (Table 1). 

**Figure 1:**
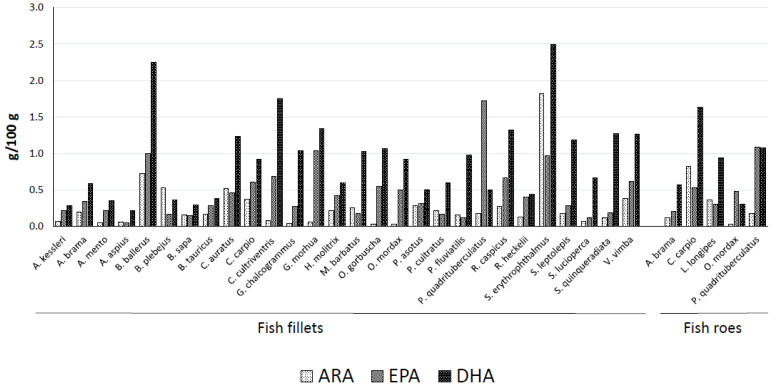
Concentrations of ARA, EPA, and DHA in the fillets and roes of dry-salted fishes analyzed in this work.

The concentrations of ARA, EPA, and DHA in fish fillets and roes are shown in Figure 1. The fillets of *S. erythrophthalmus* contain the highest amounts of ARA and DHA (1.82 and 2.49 g/100 g), which is a relevant fact due to the importance of both PUFAs for the development and performance of the central nervous system [4]. Such high DHA percentages were expected since this species contains 17.94% DHA of total FAs by wet weight [17]. However, DHA percentages for species reported here, e.g., *S. erythrophthalmus* (10.8%) and *C. carpio* (11.7%), were lower than those reported in the fresh state by [17] (17.7 and 13.98%, respectively), suggesting that the dry-salting process can induce a reduction in this highly unsaturated PUFA due to oxidative processes. However, for inland fish *C. carpio*, the DHA percentage obtained in this work agrees with that reported for fish caught in the same season (11.0%) [18], and the same is true for *G. morhua*, for which the DHA percentage obtained in this work (23.1%) agrees with that reported previously [19]. Therefore, the FA percentages of the various dry-salted fish, especially the percentages of highly unsaturated FAs, not only depend on the percentages inherent to fillets in the fresh state, which depend in turn on several factors such as diet and catch season, but also strongly depend on various factors inherent to the production process carried out in each case. 

*S. erythrophthalmus* contains DHA at amounts similar to dry-salted mackerel detailed by the USDA Nutrient Database, 2.96 g/100 g DHA, while the ARA proportion obtained here (7.9) was higher than that of mackerel (0.258 g/100 g). *S. erythrophthalmus* also provides a high amount of EPA, namely 0.97 g/100 g, which is approximately half of that reported for mackerel (1.62 g/100 g). Interestingly, the roes of this species contain both EPA and DHA (1.01 and 2.28 g/100 g), as well as good amounts of ARA (0.78 mg/100 g). This means that the consumption of just 14.4 g of fillets and 15.2 g of roes of *S. erythrophthalmus* provides the recommended daily intake of 500 mg of EPA + DHA. Dry-salted *B. ballerus* fillets also contain good amounts of ARA, EPA, and DHA (0.72, 0.99, and 2.25 mg/100 g), and therefore an intake of 15.4 g of such fillets provides the critical amount of 500 mg EPA + DHA for preventing CVD. Other samples providing 500 mg EPA + DHA al low intakes were the fillets of *C. cultriventris* (20.5 g), *G. morhua* (21.0 g), and *P. quadrituberculatus* (22.4 g) and the roes of *C. carpio* (23.0 g) and *P. quadrituberculatus* (23.1 g). In contrast, higher portions are needed to achieve 500 mg EPA + DHA when consuming the fillets of *A. kessleri* (100.9 g), *B. sapa* (112.3 g), and *A. aspius* (183.3 g). 

The fact that DHA is more abundant than EPA in most samples is relevant because of its critical role in the development and performance of the nervous and visual systems, as well as in the modulation of neuroinflammation [4]. 

### 4.3. Nutritional Quality Indices for Fatty Acids

The nutritional quality indices for fatty acids are detailed in Supplementary Table S2. The PUFA/SFA ratio is one of the indices traditionally used to assess the nutritional quality of the lipid fraction of foods, and values higher than 0.4 are desirable for decreasing CVD risk [20]. Most values in the current work fit within the range reported previously for other fish species [13]. However, the relationship between SFA intake and an increase in the risk of CVD is unclear, and other nutritional indices have been recently used to assess the nutritional quality of the lipid fraction of foods, such as AI, TI, HH, and FLQ [13].

The *n*-6/*n*-3 PUFA ratio is used for the nutritional assessment of lipids. Considering that *n*-6 and *n*-3 PUFAs are the metabolic precursors of proinflammatory and anti-inflammatory lipid mediators, respectively, an excessive intake of *n*-6 PUFAs could lead to inflammatory diseases. Therefore, the regular intake of foods having a low *n*-6/*n*-3 ratio helps to balance the proportion of both types of PUFAs and contributes to preventing or alleviating inflammatory diseases. A recent systematic review and meta-analysis study reported that a diet having a low *n*-6/*n*-3 PUFA ratio could significantly decrease the serum concentration of inflammatory markers such as tumor necrosis factor α (TNF-α) and interleukin 6 (IL-6) [21].

The AI is the ratio between those SFAs considered proatherogenic and the unsaturated FAs (UFAs), i.e., MUFAs and PUFAs, which are considered antiatherogenic. AI values lower than 1.5 are desirable, and all analyzed samples fulfilled this criterion. AI values commonly reported for fish ranged between 0.21 and 1.41 [13]. 

The TI estimates the thrombogenic potential of FAs contained in foods and is calculated as the ratio between SFAs (14:0, 16:0, and 18:0) and UFAs, although it gives more weight to *n*-3 PUFAs, which are recognized as cardiovascular health-promoting PUFAs. TI values are interpreted as the lower the value, the lower the thrombogenic risk, and values of TI < 1.15 are considered beneficial for cardiovascular health [22]. All samples showed TI values lower than 0.60, which agrees with previous reports for fish (0.14–0.87) [13].

The HH ratio is also used as a reference to estimate the potential of a given food to decrease the risk of CVD related to the metabolism of cholesterol and is considered more reliable than the PUFA/SFA ratio [13]. The higher the HH ratio, the better the protective effect against CVD. Reported HH ratios for various fish species are in the range of 1.5-3.0 [13,23]. Most HH values were within this range, highlighting the high value of *B. tauricus* (4.85).

The FLQ index is used to show EPA + DHA proportions among total FAs in marine foods. Therefore, a high FLQ index means a protective effect against the risk of CVD. Some samples showed FLQ values higher than 25 (EPA + DHA > 25%), and therefore such samples exert a high CVD prevention effect. However, the consumption of salted–dried fishes must be moderated because of their high sodium content.

According to nutritional lipid quality indices (PUFA/SFA ≥ 1.0, *n*-6/*n*-3 ratio ≤ 0.25, AI ≤ 0.40, TI ≤ 0.30, HH ≥ 2.5, and FLQ ≥ 25), some samples can be classified as excellent for decreasing the risk of CVD. These are the fillets of *A. brama*, *G. morhua*, *P. fluviatilis*, *S. lucioperca*, *S. quinqueradiata*, and *P. quadrituberculatus*, as well as the roes of *P. quadrituberculatus*. Two of these species are from marine waters (*G. morhua* and *S. quinqueradiata*), and four are from inland waters (*A. brama*, *P. fluviatilis*, *S. lucioperca*, and *P. quadrituberculatus*). 

The expected high sodium content of the samples analyzed leads to the selection of those fulfilling the recommended daily intake of 500 mg EPA + DHA through reduced consumption. In this regard, an amount less than 25 g of *G. morhua* and *P. quadrituberculatus* can provide this recommended intake.

### 4.4. Tocol and Squalene Contents

Vitamin E is the generic name given to eight isoforms grouped into four T’s and four T_3_’s, which are mainly found in vegetable oils and nuts. These compounds are produced only by photosynthetic organisms and therefore are essential for humans. Vitamin E is a lipophilic antioxidant that may have a role in the prevention or amelioration of cardiovascular and aging-related diseases such as neurological disorders, having anticancer and anti-inflammatory properties [24]. Among all isoforms of vitamin E, α-T is the most bioavailable one due to its high affinity with the hepatic α-T transfer protein (α-TPP). The bioavailability of T_3_’s is lower than that of α-T, and among them, the α isoform is the one with the highest bioavailability. When the food matrix has a low level of α-T and high levels of α-T_3_ there is an increased absorption of the last compound [24]. 

Previous reports on α-T for fillets showed similar amounts between fishes from marine (7.5–26.8 µg/g) and inland (6.6–26.3 µg/g) waters [25]. α-T values for *A. brama*, *G. morhua*, and *P. fluviatilis* (0.19, 0.76, and 0.54 mg/100 g, respectively) obtained in the current study were lower than those reported for these fish in the fresh condition (2.91, 1.05, and 1.50 mg/100 g, respectively) [25], and this fact might be due to this vitamin being partially degraded in the dry-salting process. Another study on the T profiles of the fillets of marine and inland water fishes reported no differences for fishes depending on the water type [26]. These authors reported 2.96 and 5.15 mg/100 g of total T’s for *M. barbatus* and *P. fluviatilis* respectively, whereas in the current work, *M. barbatus* and *P. fluviatilis* contained 0.16 and 0.54 mg/100 g, thus reinforcing what was previously suggested regarding the dry-salting process decreasing tocol amounts in fishes. Lower amounts of α-T were detected in the fillets of *M. barbatus* (Fam. Mullidae) and *O. mordax* (Fam. Osmeridae). For dry-salted mackerel, the USDA Nutrient Database indicates 2.38 mg/100 g [16], which is a value located at the top of the data range obtained here.

Overall, roes, especially those of fish belonging to families Cyprinidae and Pleuronectidae, seem to be a better source of T’s than fillets. For the roes of three dry-salted fishes, 8.9 (*Coregonus albula*), 5.04 (*Cuplea barengus membras*), and 15.37 mg/100 g (*Coregonus* spp.) were reported [25], which are values in good agreement with the highest values found here.

T_3_’s have been much less explored than T’s in fish. In the current work, it was only possible to quantify small amounts of α-T_3_ in two roes and γ-T_3_ in two fillets. Such small amounts of T_3_’s in fish have been previously reported [25,27].

α-T is the only component of the unsaponifiable fraction for which an adequate intake (AdI) for the population has been provided, which was set at 11 and 13 mg/day for adult females and males [6]. For calculations, the usual serving size for dry-salted fish consumption (150 g) was taken from [28]. The consumption of fillets and roes of dry-salted fish provides very different amounts of VEA. For instance, consumption of ~176 and ~208 g (for males and females) of the roes of *P. quadrituberculatus* would be enough to fulfill the AdI for VEA, which is a little more than the serving size for this food type. As for fillets, the same would be achieved through the consumption of ~346 and ~409 g of *P. quadrituberculatus*, which is approximately twice the usual serving size of salted–dried fish. However, although VEA values of roes are on average higher than those of fillets, most species display values far from these figures; thus, other VEA-rich foods are needed to fulfill daily nutritional requirements.

According to Regulations EC No. 1924/2006 and EU No. 1169/2011 (European Parliament and Council of the European Union, 2006, 2011) on nutrition and health claims made on foods, the nutrition claim “source of vitamin E” can only be applied to foods containing at least a significant amount of the vitamin under consideration, which corresponds to 15% of the AdI [29,30]. For vitamin E, the requirements were set only for α-T at 12 mg per 100 g; therefore, the content of this vitamin should be set under 1.8 mg/100 g. The fillets of two species (*S. erythrophthalmus* and *P. quadrituberculatus*) and most roes (especially the ones of Cyprinidae and Pleuronectidae species) fulfill α-T requirements. Thus, the roes of dry-salted fishes could be considered as vitamin E sources in most cases according to EU regulations.

Sq is a terpene that is naturally available in animal and vegetal sources and has cardioprotective, antioxidant, antibacterial, and anticarcinogenic properties [11]. A previous work reported Sq amounts in raw muscle from freshwater fishes caught in the Czech Republic; among others, *C. carpio*, *H. molitrix*, *A. brama*, *P. fluviatilis*, *S. erythrophthalmus*, and *A. aspius* were analyzed [31]. These authors reported Sq contents between 0.362 and 0.861 mg/100 g, whereas in the current study, values ranged from undetectable amounts to 0.39 mg/100 g for the same species. In a first approximation, higher Sq amounts could be expected in the dry-salted samples when compared to fresh samples. However, Sq is a molecule prone to oxidation due to its high degree of unsaturation, and the dry-salting process probably reduces the total amount detected in the fresh state in various fish species. 

The estimated daily intake of Sq in humans ranges between 30 and 400 mg [32]. In this regard, the richest sources of Sq analyzed in this work were the fillets of *C. cultriventris*, *S. quinqueradiata*, and *S. leptolepis*, which provide 1.83, 1.54, and 1.26 mg/100 g of Sq. Consequently, it can be concluded that the Sq supply of dry-salted fish analyzed here is quite small.

### 4.5. Considerations about Salt Content of the Analyzed Fish

Efforts have been made to reduce the sodium content of food products in the European Union via food reformulation in various industries. The World Health Organization (WHO) recommends <5 g/day of dietary salt intake (<2 g/day sodium) and provides an internationally accepted baseline for reformulation efforts. However, most Europeans continue to consume levels of salt above the recommended limit [33]. In this regard, following criteria set by the Codex Alimentarius standard for salted Atlantic herring and salted sprat, according to the process carried out in the industry, the various fishes analyzed here can be classified as lightly salted fish, in which the salt content in the fish muscle in water phase is above 4 g/100 g and below or equal to 10 g salt/100 g [34]. According to this work, a consumption of ~25 g of *G. morhua* and *P. quadrituberculatus* can provide the recommended daily intake of 500 mg EPA + DHA, and considering their salt content between 3 and 6 g/100 g, as previously explained, a total salt intake of ~0.7–1.5 g is expected through their consumption. This amount would not greatly conflict with the recommended daily sodium intake set by the WHO.

## 5. Conclusions

Overall, the fillets and roes of dry-salted fish analyzed in this work are highlighted due to their high concentrations of conditionally essential PUFAs, i.e., ARA, EPA, and DHA. In most cases, DHA percentages were higher than EPA percentages, while some species constitute excellent sources of both PUFAs. Considering the expected high sodium content of the fish analyzed, those fulfilling the recommended daily intake of 500 mg of EPA + DHA through reduced consumption should be focused on for consumption. Interestingly, reduced consumption of most species could be enough to fulfil the recommended daily intake of long-chain *n*-6 and *n*-3 PUFAs. Moreover, the fillets and roes analyzed here contain high concentrations of α-T, which was the most prominent tocol found in all organs and species, and most samples display small amounts of Sq. Interestingly, the roes of most Cyprinidae species could be considered as sources of vitamin E. Future actions regarding this type of food should be aimed at designing dry-salting processes able to avoid the loss of bioactive compounds (T’s, T_3_’s, and Sq) in the resulting products.

## Figures and Tables

**Table 1:** Data on analyzed samples.

Label	Species	Common Name	Local Name	Catching Area	Catching Month	Average Body Size	
						Length, cm	Weight, g
**Order *Clupeiformes***							
Family: *Clupeidae*							
1	*Alosa kessleri* (Grimm, 1887)	Caspian anadromous shad	Sel’d-chernospinka, beshenka	Volga–Caspian fishery basin, Northern fishery region, fishing areas of the Volga delta, Russian Federation	June	30–40	500–960
2	*Clupeonella cultriventris* (Nordmann, 1840)	Black and Caspian Sea sprat	Tyul’ka	Volga–Caspian fishery basin, Southern fishery region, Caspian Sea, Russian Federation	July	6–10	70–150
**Order *Cypriniformes***							
Family *Cyprinidae*							
3	*Abramis brama* (Linnaeus, 1758)	Freshwater bream	Lesh	Volga–Caspian fishery basin, Northern fishery region, fishing areas of the Volga delta, Russian Federation	May	30–40	500–650
4	*Alburnus mento* (Heckel, 1836)	Bleak	Chernomorsko-azovskaya shemaya	Azovo-Chernomorsky fishery basin, Veselovsky reservoir, left tributary of the river Don, Russian Federation	April	19–22	100–300
5	*Aspius aspius* (Linnaeus, 1758)	Asp	Zhereh, belest’	Volga–Caspian fishery basin, Southern fishery region, Caspian Sea, Russian Federation	May	33–50	1200–1500
6	*Ballerus ballerus (Abramis ballerus)* (Linnaeus, 1758)	Zope or blue bream	Sinetc	Volga–Caspian fishery basin, Northern fishery region, fishing areas of the Volga delta, Russian Federation	June	24–28	300–400
7	*Ballerus sapa* (Pallas, 1814)	White-eye bream	Beloglazka, sopa	Volga–Caspian fishery basin, Northern fishery region, Kuibyshev reservoir, Russian Federation	May	21–27	150–250
8	*Barbus tauricus* (Kessler, 1877)	Crimean barbel	Usach krimskiy	Azovo-Chernomorsky fishery basin, Kuban River, Russian Federation	July	15–36	900–1100
9	*Blicca bjoerkna* (Linnaeus, 1758)	White bream	Gustera	Volga–Caspian fishery basin, Northern fishery region, Kuibyshev reservoir, Russian Federation	July	20–24	100–200
10	*Carassius auratus* (Linnaeus, 1758)	Goldfish	Zolotaya ribka, karas kitayskiy, srebryaniy karas	Volga–Caspian fishery basin, Northern fishery region, Kazan Bay of the Kuibyshev Reservoir, Russian Federation	May	16–24	450–650
11	*Cyprinus carpio* (Linnaeus, 1758)	Common carp	Sazan, karp obiknovenniy	Volga–Caspian fishery basin, Southern fishery region, Caspian Sea, Russian Federation	June	30–50	350–600
12	*Hypophthalmichthys molitrix* (Valenciennes, 1844)	Silver carp	Tolstolobik	Russian Far East fishery basin, lake Khanka/Xinkaihu river basin Amur, Russian Federation	August	30–60	2000–5000
13	*Pelecus cultratus* (Linnaeus, 1758)	Ziege, sichel, sabre carp, or sabrefish	Chehon’	Volga–Caspian fishery basin, Southern fishery region, Caspian Sea, Russian Federation	June	30–50	700–1100
14	*Rutilus caspicus* (Yakovlev, 1870)	Caspian roach	Vobla	Volga–Caspian fishery basin, Southern fishery region, Caspian Sea, Russian Federation	May	22–35	500–800
15	*Rutilus heckelii* (Nordmann, 1840)	Roach	Taran, taranka	Azovo-Chernomorsky fishery basin, Sea of Azov, Russian Federation	April	15–25	400–1000
16	*Scardinius erythrophthalmus* (Linnaeus, 1758)	Common rudd	Krasnoperka, krasnoglazka	Azovo-Chernomorsky fishery basin, Sea of Azov, Russian Federation	May	20–25	700–1200
17	*Vimba vimba* (Linnaeus, 1758)	Vimba bream	Ribec obiknovenniy	Volga–Caspian fishery basin, Southern fishery region, Caspian Sea, Russian Federation	May	23–34	300–500
**Order *Gadiformes***							
Family *Gadidae*							
18	*Gadus chalcogrammus* (Pallas, 1814)	Alaska pollock	Mintay	Russian Far East fishery basin, West Bering Sea zone, Bering Sea, Russian Federation	January	50–70	2500–5000
19	*Gadus morhua* (Linnaeus, 1758)	Atlantic cod	Treska	Northern fishery basin, Barents Sea, Russian Federation	October	55–65	200–1100
Family *Moridae*							
20	*Laemonema longipes* (Schmidt, 1938)	Longfin codling	Lemonema	Russian Far East fishery basin, region of the South Kuril, The Russian Kuril Islands, Russian Federation	August	49–53	500–850
Family *Percidae*							
21	*Perca fluviatilis* (Linnaeus, 1758)	European perch	Okun’ rechnoy	Volga–Caspian fishery basin, Northern fishery region, Kuibyshev reservoir, Russian Federation	November	22–30	300–500
**Order *Osmeriformes***							
Family *Osmeridae*							
22	*Osmerus mordax* (Mitchill, 1814)	Rainbow smelt	Koryshka	Russian Far East fishery basin, Sea of Okhotsk zone, West Kamchatka Subzone, Shelikhov Bay, Russian Federation	May	25–31	140–300
23	*Sander lucioperca* (Linnaeus, 1758)	Pike-perch	Sudak	Volga–Caspian fishery basin, Northern fishery region, Kuibyshev reservoir, Russian Federation	August	32–40	870–930
**Order *Perciformes***							
Family *Carangidae*							
24	*Selaroides leptolepis* (Cuvier, 1833)	Yellowstripe scad	Selar, zheltiy polosatik	Gulf of Tonkin, Vietnam	November	10–22	300–600
25	*Seriola quinqueradiata* (Temminck and Schlegel, 1845)	Japanese amberjack	Lakedra, zheltohvost	Russian Far East fishery basin, Sea of Japan zone, The Peter the Great Gulf, Russian Federation	August	800–1000	>9000
Family *Mullidae*							
26	*Mullus barbatus* (Linnaeus, 1758)	Red mullet	Barabul’ka	Azovo-Chernomorsky fishery basin, Kerch Strait, Russian Federation	April	10–20	40–70
**Order *Pleuronectiformes***							
Family *Pleuronectidae*							
27	*Pleuronectes quadrituberculatus* (Pallas, 1814)	Alaska plaice	Kambala zheltobryuhaya (chetirehbugorchataya)	Russian Far East fishery basin, Sea of Okhotsk zone, West Kamchatka Subzone, Russian Federation	August	25–40	600–1100
**Order *Salmoniformes***							
Family *Salmonidae*							
28	*Oncorhynchus gorbuscha* (Walbaum, 1792)	Pink salmon	Gorbusha	Russian Far East fishery basin, East Kamchatka zone, Storozh river, Russian Federation	July	43–50	1000–1300
**Order *Siluriformes***							
Family *Siluridae*							
29	*Parasilurus asotus* (Linnaeus, 1758)	Amur catfish	Som	Russian Far East fishery basin, lake Khanka/Xinkaihu river basin Amur, Russian Federation	May	50–60	1000–1500

**Table 2:** Moisture, fatty acid profile, and total fatty acid content of sampled fishes and roes. Data are shown as mean value ± SD (n = 5).

Species	Fatty Acids (FA% of Total FAs) ^†,‡^																			Moisture (g/100 g)	Total FAs (g/100 g)
	14:0	15:0	16:0	17:0	18:0	20:0	16:1*n*-7	18:1*n*-9	18:1*n*-7	20:1*n*-9	24:1*n*-9	18:2*n*-6	18:3*n*-3	18:4*n*-3	20:4*n*-6	20:4*n*-3	20:5*n*-3	22:5*n*-3	22:6*n*-3		
**Fillets**		**Family *Carangidae***																			
*S. leptolepis*	1.6 ± 0.3 ^efg^	n.d.	21.6 ± 1.1 ^lmnop^	1.3 ± 0.2 ^fgh^	14.2 ± 0.2 ^p^	n.d.	2.7 ± 0.3 ^b^	7.7 ± 0.4 ^bc^	2.1 ± 0.1 ^bcde^	n.d.	2.6 ± 0.2 ^j^	1.9 ± 0.4 ^ij^	0.9 ± 0.0 ^g^	n.d.	3.8 ± 0.1 ^ij^	0.8 ± 0.0 ^h^	6.0 ± 0.0 ^ij^	4.3 ± 0.3 ^no^	25.3 ± 0.9 ^m^	16.3 ± 0.1 ^f^	4.7 ± 0.2 ^h^
*S. quinqueradiata*	0.9 ± 0.1 ^ab^	0.7 ± 0.0 ^ef^	18.6 ± 0.3 ^efgh^	1.4 ± 0.1 ^gh^	13.9 ± 0.4 ^p^	n.d.	1.2 ± 0.1 ^a^	9.2 ± 0.5 ^cde^	1.9 ± 0.3 ^bcd^	n.d.	n.d.	n.d.	n.d.	n.d.	3.2 ± 0.3 ^gh^	n.d.	5.0 ± 0.4 ^fg^	7.1 ± 0.4 ^s^	34.4 ± 1.0 ^p^	17.4 ± 0.1 ^h^	3.7 ± 0. 1^def^
		**Family *Clupeidae***																			
*A. kessleri*	2.5 ± 0.3 ^jklm^	0.6 ± 0.0 ^de^	17.0 ± 0.5 ^de^	0.8 ± 0.1 ^cde^	3.5 ± 0.3 ^bc^	1.6 ± 0.2 ^e^	12.4 ± 0.7 ^q^	31.3 ± 0.8 ^u^	4.0 ± 0.1 ^jkl^	1.3 ± 0.0 ^fgh^	n.d. ^a^	1.1 ± 0.1 ^defg^	0.8 ± 0.0 ^fg^	n.d.	1.9 ± 0.1 ^d^	n.d.	5.4 ± 0.2 ^ghi^	3.1 ± 0.2 ^hij^	7.3 ± 0.4 ^abc^	14.0 ± 0.1 ^bc^	3.9 ± 0.0 ^f^
*C. cultriventris*	5.5 ± 0.2 ^q^	0.3 ± 0.0 ^b^	17.3 ± 0.6 ^de^	3.3 ± 0.2 ^j^	4.2 ± 0.3 ^cd^	0.2 ± 0.0 ^ab^	16.2 ± 0.1 ^s^	30.0 ± 1.5 ^u^	2.3 ± 0.1 ^cde^	0.2 ± 0.0 ^ab^	0.6 ± 0.1 ^de^	0.4 ± 0.2 ^ab^	3.1 ± 0.0 ^l^	1.0 ± 0.1 ^d^	0.4 ± 0.0 ^a^	0.2 ± 0.0 ^c^	3.5 ± 0.0 ^cd^	0.9 ± 0.1 ^b^	8.9 ± 0.2 ^de^	16.7 ± 0.2 ^g^	19.7 ± 0.2 ^s^
		**Family *Cyprinidae***																			
*A. brama*	2.1 ± 0.2 ^ghij^	0.5 ± 0.0 ^cd^	16.2 ± 0.4 ^cd^	0.7 ± 0.0 ^bcd^	4.8 ± 0.3 ^de^	3.6 ± 0.1^i^	4.7 ± 0.2 ^d^	24.8 ± 0.5 ^r^	2.9 ± 0.0 ^gh^	0.8 ± 0.0 ^d^	n.d. ^a^	0.9 ± 0.1 ^cde^	0.5 ± 0.0 ^cde^	n.d.	5.2 ± 0.2 ^l^	n.d.	9.1 ± 0.1 ^qr^	4.6 ± 0.1 ^op^	15.4 ± 0.3 ^h^	12.7 ± 0.1 ^a^	3.8 ± 0.0 ^ef^
*A. mento*	4.3 ± 0.1 ^op^	0.9 ± 0.0 ^gh^	20.9 ± 1.3 ^klmn^	0.7 ± 0.0 ^bcd^	4.5 ± 0.2 ^d^	n.d.	4.7 ± 0.1 ^d^	16.9 ± 1.9 ^no^	4.1 ± 0.7 ^klm^	0.4 ± 0.0 ^bc^	0.4 ± 0.0 ^bc^	2.0 ± 0.0 ^j^	4.7 ± 0.6 ^n^	3.2 ± 0.2 ^i^	1.5 ± 0.2 ^c^	0.9 ± 0.0 ^i^	6.4 ± 0.1 ^jkl^	1.9 ± 0.2 ^de^	10.4 ± 0.3 ^ef^	14.6 ± 0.1 ^d^	3.4 ± 0.2 ^cde^
*A. aspius*	2.7 ± 0.1 ^lm^	0.9 ± 0.0 ^gh^	22.4 ± 0.6 ^nopqr^	0.8 ± 0.0 ^cde^	6.5 ± 0.3 ^i^	3.4 ± 0.3^i^	9.4 ± 0.3 ^n^	28.3 ± 0.2 ^t^	3.8 ± 0.2 ^ijk^	1.1 ± 0.2 ^e^	0.6 ± 0.0 ^de^	0.8 ± 0.1 ^bcde^	0.5 ± 0.0 ^cde^	n.d.	1.9 ± 0.1 ^d^	n.d.	1.7 ± 0.3 ^a^	1.1 ± 0.3 ^b^	7.1 ± 0.3 ^abc^	12.5 ± 0.3 ^a^	3.1 ± 0.1 ^c^
*B. ballerus*	1.6 ± 0.2 ^def^	0.9 ± 0.0 ^gh^	23.2 ± 1.4 ^pqrs^	n.d.	6.4 ± 0.4 ^hi^	n.d.	7.1 ± 0.0 ^k^	14.5 ± 0.4 ^kl^	3.5 ± 0.0 ^ij^	n.d.	n.d.	1.4 ± 0.1 ^fghi^	1.9 ± 0.2 ^i^	n.d.	6.4 ± 0.4 ^m^	1.1 ± 0.0 ^k^	8.8 ± 0.2 ^pq^	2.3 ± 0.1 ^ef^	19.9 ± 1.6 ^k^	17.3 ± 0.2 ^h^	11.3 ± 0.3 ^q^
*B. bjoerkna*	3.7 ± 0.3 ^n^	0.9 ± 0.0 ^gh^	17.8 ± 0.7 ^def^	0.9 ± 0.1 ^def^	9.6 ± 0.2 ^mn^	n.d.	8.7 ± 0.4 ^m^	22.9 ± 0.4 ^q^	3.9 ± 0.1 ^ijkl^	4.7 ± 0.2 ^m^	n.d.	0.7 ± 0.0 ^bcd^	0.5 ± 0.1 ^cde^	n.d.	3.4 ± 0.3 ^hi^	0.6 ± 0.0 ^f^	5.8 ± 0.4 ^hij^	3.5 ± 0.1 ^jkl^	7.8 ± 0.2 ^cd^	17.2 ± 0.2 ^h^	4.9 ± 0.2 ^hi^
*B. sapa*	1.3 ± 0.2 ^bcde^	0.6 ± 0.1 ^de^	23.1 ± 0.8 ^pqrs^	0.6 ± 0.1 ^bc^	10.2 ± 0.3 ^n^	n.d.	5.5 ± 0.6 ^efgh^	11.9 ± 1.0 ^hi^	2.9 ± 0.1 ^gh^	n.d.	n.d.	1.1 ± 0.2 ^cdefg^	0.7 ± 0.0 ^efg^	n.d.	9.3 ± 0.1 ^p^	n.d.	8.7 ± 0.4 ^pq^	4.2 ± 0.2 ^no^	17.5 ± 1.0 ^i^	18.0 ± 0.1 ^i^	1.7 ± 0.4 ^a^
*B. tauricus*	1.5 ± 0.3 ^def^	0.7 ± 0.0 ^ef^	10.3 ± 0.2 ^a^	2.6 ± 0.1 ^i^	4.9 ± 0.2 ^de^	2.3 ± 0.2 ^g^	7.1 ± 0.6 ^k^	16.7 ± 0.3 ^n^	3.4 ± 0.1 ^hi^	1.5 ± 0.0 ^h^	n.d.	13.5 ± 0.2 ^n^	1.9 ± 0.1 ^i^	n.d.	8.9 ± 0.2 ^o^	n.d.	2.8 ± 0.1 ^b^	3.9 ± 0.1 ^lmn^	6.1 ± 0.2 ^a^	13.8 ± 0.2 ^b^	5.9 ± 0.2 ^k^
*C. auratus*	1.1 ± 0.1 ^abcd^	0.5 ± 0.0 ^cd^	21.8 ± 1.3 ^lmnop^	0.6 ± 0.1 ^bc^	6.2 ± 0.5 ^fghi^	n.d.	8.0 ± 0.1 ^l^	11.8 ± 0.5 ^ghi^	2.5 ± 0.0 ^fg^	n.d.	0.6 ± 0.0 ^de^	1.7 ± 0.1 ^ij^	0.6 ± 0.1 ^def^	n.d.	7.7 ± 0.4 ^n^	0.7 ± 0.1 ^g^	6.8 ± 0.3 ^kl^	2.5 ± 0.2 ^fg^	18.2 ± 0.7 ^ij^	15.1 ± 0.2 ^e^	6.8 ± 0.2 ^l^
*C. carpio*	1.4 ± 0.0 ^cdef^	2.1 ± 0.3 ^j^	14.1 ± 0.6 ^b^	n.d.	15.0 ± 0.3 ^q^	3.1 ± 0.4 ^h^	6.3 ± 0.2 ^i^	9.9 ± 0.6 ^def^	4.1 ± 0.1 ^klm^	1.2 ± 0.0 ^efg^	3.2 ± 0.2 ^k^	1.2 ± 0.0 ^efgh^	2.0 ± 0.2 ^i^	n.d.	4.7 ± 0.1 ^k^	n.d.	7.7 ± 0.2 ^mno^	5.7 ± 0.3 ^r^	11.7 ± 0.2 ^fg^	18.9 ± 0.2 ^k^	7.9 ± 0.1 ^no^
*H. molitrix*	6.0 ± 0.5^r^	0.8 ± 0.1 ^fg^	21.1 ± 1.1 ^klmno^	0.8 ± 0.0 ^cde^	7.5 ± 0.0 ^j^	0.3 ± 0.1 ^b^	8.9 ± 0.3 ^mn^	26.6 ± 0.8 ^s^	2.1 ± 0.2 ^bcde^	1.2 ± 0.2 ^ef^	n.d.	3.7 ± 0.4 ^l^	0.3 ± 0.0 ^bc^	1.9 ± 0.1 ^f^	2.6 ± 0.3 ^ef^	0.4 ± 0.0 ^d^	5.2 ± 0.1 ^gh^	1.6 ± 0.1 ^cd^	7.3 ± 0.7 ^abcd^	19.3 ± 0.2 ^l^	8.2 ± 0.4 ^o^
*P. cultratus*	1.8 ± 0.1 ^fghi^	0.6 ± 0.0 ^de^	24.1 ± 0.9 ^rs^	0.7 ± 0.1 ^bcd^	10.1 ± 1.5 ^mn^	0.9 ± 0.1 ^cd^	6.4 ± 0.1 ^ij^	33.7 ± 1.2 ^v^	2.2 ± 0.4 ^bcde^	1.1 ± 0.1 ^ef^	0.5 ± 0.0 ^cd^	1.0 ± 0.1 ^cdef^	0.5 ± 0.0 ^cde^	n.d.	2.7 ± 0.1 ^f^	n.d.	2.1 ± 0.2 ^a^	1.8 ± 0.3 ^d^	7.7 ± 0.4 ^bcd^	16.3 ± 0.1 ^f^	7.8 ± 0.1 ^no^
*R. caspicus*	1.6 ± 0.3 ^efg^	0.6 ± 0.0 ^de^	20.8 ± 1.0 ^jklmn^	n.d.	5.5 ± 0.2 ^ef^	n.d.	7.0 ± 0.3 ^jk^	18.5 ± 0.7 ^p^	3.7 ± 0.1 ^ijk^	2.1 ± 0.1 ^ij^	n.d.	0.6 ± 0.0 ^bc^	n.d. ^a^	n.d.	4.0 ± 0.3 ^j^	n.d.	9.7 ± 0.4 ^r^	3.7 ± 0.2 ^klm^	19.1 ± 0.1^jk^	15.1 ± 0.2 ^e^	6.9 ± 0.1 ^l^
*R. heckelii*	2.6 ± 0.2 ^klm^	0.7 ± 0.1 ^ef^	20.7 ± 1.4 ^jklmn^	0.8 ± 0.0 ^cde^	6.2 ± 0.1 ^ghi^	1.1 ± 0.0 ^d^	11.3 ± 0.4 ^p^	23.4 ± 0.4 ^qr^	5.7 ± 0.1 ^qr^	2.3 ± 0.1 ^j^	n.d.	2.6 ± 0.2 ^k^	1.4 ± 0.2 ^h^	n.d.	2.3 ± 0.2 ^de^	n.d.	7.1 ± 0.4 ^lm^	2.6 ± 0.3 ^fgh^	7.8 ± 0.2 ^cd^	17.2 ± 0.2 ^h^	5.7 ± 0.3 ^jk^
*S. erythrophthalmus*	1.7 ± 0.1 ^efgh^	n.d.	22.1 ± 0.8 ^mnopq^	5.1 ± 0.3 ^k^	4.4 ± 0.0 ^d^	n.d.	5.9 ± 0.0 ^ghi^	13.6 ± 0.0 ^jk^	4.1 ± 0.3 ^klm^	1.3 ± 0.0 ^fgh^	n.d.	8.1 ± 0.4 ^m^	5.8 ± 0.0 ^o^	n.d.	7.9 ± 0.4 ^n^	1.0 ± 0.1 ^j^	4.2 ± 0.0 ^e^	n.d.	10.8 ± 0.6 ^f^	16.2 ± 0.2 ^f^	23.1 ± 0.4 ^t^
*V. vimba*	1.7 ± 0.1 ^efgh^	0.6 ± 0.0 ^de^	19.5 ± 0.3 ^fghij^	1.0 ± 0.1 ^efg^	5.8 ± 0.2 ^fgh^	0.8 ± 0.1 ^c^	7.5 ± 0.1 ^kl^	18.8 ± 1.2 ^p^	4.6 ± 0.3 ^mno^	1.3 ± 0.1 ^fgh^	n.d.	3.8 ± 0.1 ^l^	4.0 ± 0.2 ^m^	n.d.	3.9 ± 0.2 ^j^	0.7 ± 0.0 ^g^	6.2 ± 0.6 ^jk^	3.1 ± 0.2 ^hij^	12.8 ± 0.2^g^	14.2 ± 0.2 ^c^	9.9 ± 0.3 ^p^
		**Family *Gadidae***																			
*G. chalcogrammus*	1.3 ± 0.2 ^bcde^	n.d.	24.7 ± 0.6 ^st^	n.d.	5.6 ± 0.1 ^fg^	n.d.	1.6 ± 0.2 ^a^	10.4 ± 0.5 ^efg^	2.5 ± 0.2 ^efg^	4.5 ± 0.4 ^m^	1.2 ± 0.3 ^h^	3.0 ± 0.2 ^j^	0.8 ± 0.0 ^fg^	n.d.	1.2 ± 0.1 ^bc^	0.9 ± 0.1 ^i^	8.3 ± 0.3 ^op^	2.4 ± 0.2 ^f^	31.5 ± 0.7 ^o^	16.4 ± 0.1 ^f^	3.3 ± 0.0 ^cde^
*G. morhua*	2.3 ± 0.2 ^ijkl^	n.d.	14.9 ± 0.1 ^bc^	n.d.	3.4 ± 0.2 ^b^	n.d.	7.0 ± 0.3 ^jk^	16.0 ± 0.5 ^lmn^	4.4 ± 0.3 ^lmno^	4.6 ± 0.3 ^m^	1.0 ± 0.1 ^g^	1.0 ± 0.1 ^cdef^	n.d. ^a^	n.d.	1.1 ± 0.1 ^bc^	n.d.	17.9 ± 0.6 ^w^	1.2 ± 0.2 ^bc^	23.1 ± 0.7^l^	15.2 ± 0.2 ^e^	5.8 ± 0.2 ^k^
		**Family *Mullidae***																			
*M. barbatus*	3.0 ± 0.2 ^m^	1.0 ± 0.1 ^h^	17.9 ± 0.4 ^defg^	1.5 ± 0.2 ^h^	12.0 ± 0.4 ^o^	n.d.	2.4 ± 0.3 ^b^	7.0 ± 0.2 ^ab^	18.0 ± 0.6 ^u^	0.6 ± 0.1 ^cd^	1.6 ± 0.2 ^i^	1.5 ± 0.1 ^ghi^	n.d.	n.d.	4.8 ± 0.1 ^kl^	n.d.	3.4 ± 0.3 ^bc^	4.1 ± 0.4 ^mn^	19.8 ± 1.0 ^k^	16.3 ± 0.3 ^f^	5.2 ± 0.3 ^ij^
		**Family *Osmeridae***																			
*O.* *mordax*	4.2 ± 0.2 ^nop^	0.3 ± 0.1 ^b^	20.3 ± 1.0 ^hijk^	0.6 ± 0.0 ^bc^	5.8 ± 0.4 ^fgh^	0.7 ± 0.1 ^c^	9.1 ± 0.5 ^mn^	12.5 ± 0.6 ^ij^	6.0 ± 0.3 ^rs^	0.4 ± 0.0 ^bc^	0.4 ± 0.0 ^bc^	0.9 ± 0.1 ^cde^	0.6 ± 0.0 ^def^	1.1 ± 0.1 ^d^	0.8 ± 0.0 ^ab^	n.d.	12.2 ± 0.5 ^s^	1.0 ± 0.2 ^b^	22.5 ± 0.4 ^l^	17.3 ± 0.1 ^h^	4.1 ± 0.2 ^fg^
*S. lucioperca*	1.4 ± 0.0 ^cdef^	0.5 ± 0.0 ^cd^	20.7 ± 1.9 ^jklmn^	0.5 ± 0.1 ^b^	6.0 ± 0.2 ^fghi^	0.3 ± 0.0 ^b^	5.1 ± 0.3 ^def^	18.2 ± 0.3 ^op^	1.9 ± 0.4 ^bc^	0.2 ± 0.0 ^ab^	0.7 ± 0.0 ^ef^	1.0 ± 0.1 ^cdef^	2.7 ± 0.3 ^k^	0.2 ± 0.0 ^b^	2.9 ± 0.3 ^fg^	0.1 ± 0.0 ^b^	5.1 ± 0.2 ^fg^	1.0 ± 0.1 ^b^	27.8 ± 0.9 ^n^	14.6 ± 0.1 ^d^	2.4 ± 0.1 ^b^
		**Family *Percidae***																			
*P. fluviatilis*	1.0 ± 0.1 ^abc^	1.3 ± 0.1 ^i^	19.6 ± 0.4 ^ghij^	1.0 ± 0.0 ^efg^	8.8 ± 0.4 ^kl^	n.d.	1.5 ± 0.2 ^a^	9.4 ± 0.4 ^def^	1.7 ± 0.1 ^ab^	n.d.	n.d. ^a^	n.d. ^a^	0.2 ± 0.0 ^ab^	n.d.	4.8 ± 0.3 ^kl^	n.d.	3.8 ± 0.2 ^cde^	7.9 ± 0.2 ^t^	30.6 ± 0.7 ^o^	17.3 ± 0.1 ^h^	3.2 ± 0.1 ^cd^
		**Family *Pleuronectidae***																			
*P. quadrituberculatus*	3.9 ± 0.1 ^no^	n.d	13.3 ± 0.5 ^b^	1.4 ± 0.2 ^gh^	2.3 ± 0.0 ^a^	n.d.	17.0 ± 0.2 ^t^	12.7 ± 1.1 ^ij^	6.5 ± 0.6 ^s^	1.4 ± 0.0 ^gh^	n.d.	0.8 ± 0.0 ^bcde^	n.d.	2.1 ± 0.1 ^g^	2.2 ± 0.0 ^de^	n.d.	21.3 ± 0.1 ^y^	3.3 ± 0.2 ^ijk^	6.2 ± 0.2 ^ab^	20.3 ± 0.1 ^m^	8.1 ± 0.1 ^o^
		**Family *Salmonidae***																			
*O. gorbuscha*	4.5 ± 0.3 ^p^	0.6 ± 0.0 ^de^	10.3 ± 0.4 ^a^	n.d.	5.5 ± 0.3 ^efg^	9.6 ± 0.4 ^j^	3.4 ± 0.2 ^c^	12.3 ± 0.4 ^hij^	1.3 ± 0.2 ^a^	4.1 ± 0.1 ^l^	1.2 ± 0.1 ^h^	1.4 ± 0.1 ^fghi^	0.9 ± 0.0 ^g^	3.0 ± 0.2 ^h^	0.5 ± 0.0 ^a^	1.3 ± 0.1 ^l^	7.6 ± 0.5 ^mn^	2.6 ± 0.3 ^fgh^	14.8 ± 0.7 ^h^	17.2 ± 0.1 ^h^	7.2 ± 0.0 ^lm^
		**Family *Siluridae***																			
*P. asotus*	2.1 ± 0.1 ^hijk^	0.6 ± 0.0 ^de^	18.0 ± 0.8 ^defg^	0.7 ± 0.1 ^bcd^	9.4 ± 0.0 ^lm^	1.4 ± 0.1 ^e^	10.1 ± 0.4 ^o^	19.7 ± 1.2 ^p^	5.4 ± 0.1 ^pq^	2.0 ± 0.0 ^i^	0.6 ± 0.0 ^de^	3.5 ± 0.3 ^l^	2.4 ± 0.2 ^j^	n.d. ^a^	4.0 ± 0.2 ^j^	0.5 ± 0.0 ^e^	4.4 ± 0.3 ^ef^	4.1 ± 0.3 ^mn^	7.0 ± 0.6 ^abc^	18.3 ± 0.1 ^j^	7.2 ± 0.3 ^lm^
**Roes**		**Family *Cyprinidae***																			
*A* *. brama*	1.6 ± 0.2 ^def^	0.9 ± 0.0 ^gh^	22.8 ± 0.3 ^opqr^	0.9 ± 0.2 ^cde^	5.5 ± 0.2 ^ef^	1.9 ± 0.0 ^f^	4.7 ± 0.1 ^d^	16.5 ± 0.7 ^mn^	3.8 ± 0.3 ^ijk^	1.5 ± 0.0 ^h^	0.7 ± 0.0 ^ef^	0.9 ± 0.0 ^cde^	1.9 ± 0.1 ^i^	n.d.	4.2 ± 0.1 ^j^	n.d. ^a^	7.1 ± 0.2 ^lm^	2.9 ± 0.0 ^ghi^	19.8 ± 0.1 ^k^	22.4 ± 0.2 ^n^	2.9 ± 0.1 ^bc^
*C. carpio*	0.9 ± 0.0 ^ab^	0.7 ± 0.1 ^ef^	20.6 ± 0.5 ^jklm^	0.7 ± 0.0 ^bcd^	5.9 ± 0.1 ^fghi^	0.8 ± 0.0 ^c^	5.4 ± 0.3 ^efg^	16.7 ± 0.3 ^n^	4.2 ± 0.2^klmn^	0.6 ± 0.0 ^cd^	0.8 ± 0.0 ^f^	1.0 ± 0.1 ^cdef^	0.4 ± 0.0 ^bcd^	n.d.	6.5 ± 0.2 ^m^	n.d. ^a^	4.2 ± 0.1 ^e^	5.5 ± 0.3 ^r^	12.9 ± 0.8 ^g^	24.4 ± 0.1 ^o^	12.7 ± 0.5 ^r^
*R. caspicus*	0.9 ± 0.0 ^ab^	0.9 ± 0.1 ^gh^	23.8 ± 1.1 ^qrs^	0.9 ± 0.0 ^def^	5.7 ± 0.1 ^fgh^	n.d.	4.9 ± 0.3 ^de^	10.8 ± 0.3 ^fgh^	4.8 ± 0.1 ^o^	0.7 ± 0.0 ^d^	n.d. ^a^	n.d. ^a^	0.8 ± 0.0 ^fg^	n.d.	4.0 ± 0.2 ^j^	0.4 ± 0.0 ^d^	13.1 ± 0.7 ^t^	3.9 ± 0.0 ^lmn^	22.6 ± 6.9 ^l^	25.1 ± 0.2 ^p^	4.6 ± 0.2 ^gh^
*S. erythrophthalmus*	0.7 ± 0.1 ^a^	0.4 ± 0.1 ^bc^	26.2 ± 1.1 ^t^	0.6 ± 0.0 ^bc^	8.2 ± 0.2 ^jk^	n.d.	6.1 ± 0.2 ^hi^	8.5 ± 0.2 ^cd^	4.7 ± 0.3 ^no^	n.d.	n.d. ^a^	2.6 ± 0.0 ^k^	2.3 ± 0.0 ^j^	n.d.	6.1 ± 0.2 ^m^	0.9 ± 0.0 ^i^	7.9 ± 0.2 ^no^	2.7 ± 0.0 ^fgh^	17.8 ± 0.4 ^ij^	20.4 ± 0.1 ^m^	12.8 ± 0.2 ^r^
		**Family *Moridae***																			
*L. longipes*	1.1 ± 0.1 ^abcd^	0.5 ± 0.1 ^cd^	23.8 ± 0.4 ^qrs^	0.7 ± 0.0 ^bcd^	8.8 ± 0.3 ^kl^	n.d.	5.6 ± 0.1 ^fgh^	18.6 ± 0.5 ^p^	6.2 ± 0.1 ^rs^	2.7 ± 0.2 ^k^	0.4 ± 0.0 ^bc^	1.6 ± 0.0 ^hij^	0.9 ± 0.0 ^g^	n.d.	4.9 ± 0.4 ^kl^	0.4 ± 0.0 ^d^	4.1 ± 0.1 ^de^	5.0 ± 0.3 ^pq^	12.5 ± 0.5 ^g^	25.4 ± 0.1 ^q^	7.5 ± 0.1 ^mn^
		**Family *Osmeridae***																			
*O. mordax*	5.8 ± 0.6 ^qr^	0.4 ± 0.0 ^bc^	19.1 ± 0.8 ^fghi^	0.6 ± 0.1 ^bc^	2.0 ± 0.2 ^a^	n.d.	13.7 ± 0.3 ^r^	15.2 ± 0.7 ^lm^	7.4 ± 0.4 ^t^	0.3 ± 0.0 ^b^	0.3 ± 0.0 ^b^	1.0 ± 0.1 ^cdef^	0.7 ± 0.0 ^efg^	1.6 ± 0.2 ^e^	1.0 ± 0.1 ^b^	0.4 ± 0.0 ^d^	16.4 ± 0.6^u^	1.1 ± 0.2 ^b^	10.5 ± 0.2 ^f^	25.6 ± 0.2 ^q^	2.9 ± 0.1 ^bc^
		**Family *Pleuronectidae***																			
*P. quadrituberculatus*	2.1 ± 0.1 ^hijk^	0.5 ± 0.0 ^cd^	17.9 ± 1.3 ^defg^	1.2 ± 0.2 ^fgh^	4.7 ± 0.1 ^d^	n.d.	4.7 ± 0.3 ^d^	5.8 ± 0.0 ^a^	4.9 ± 0.0 ^op^	0.8 ± 0.0 ^d^	n.d.	n.d.	n.d.	0.4 ± 0.0 ^c^	3.0 ± 0.2 ^fg^	n.d.	18.8 ± 0.3 ^x^	5.3 ± 0.3 ^qr^	18.5 ± 0.9 ^ijk^	22.2 ± 0.2 ^n^	5.8 ± 0.0 ^k^

^†^ Other FAs of undetermined structure accounted for 100% of total FAs; ^‡^ within each column, different superscript letters indicate significant differences among values (*p* < 0.05) according to one-way ANOVA followed by Duncan’s test; n.d.: not detected (concentrations below LOQ, as reported in Supplementary File S1).

**Table 3:** Tocol profiles and contents, VEA, and squalene (mg/100 g) of sampled fishes and roes. Data are shown as mean value ± SD (n = 5) ^a^.

	α-T	γ-T	α-T_3_	γ-T_3_	Total Tocols	VEA	Squalene
**Fillets**							
	**Family *Carangidae***						
*S. leptolepis*	0.39 ± 0.04 ^de^	n.d.	n.d.	n.d.	0.39 ± 0.0 ^de^	0.39 ± 0.4 ^de^	1.26 ± 0.04 ^p^
*S. quinqueradiata*	1.03 ± 0.08 ^k^	n.d.	n.d.	n.d.	1.03 ± 0.08 ^k^	1.03 ± 00.8 ^k^	1.54 ± 0.04 ^q^
	**Family *Clupeidae***						
*A. kessleri*	0.54 ± 0.01 ^g^	n.d.	n.d.	n.d.	0.54 ± 0.01 ^g^	0.54 ± 0.0 ^g^	0.31 ± 0.10 ^hi^
*C. cultriventris*	0.53 ± 0.06 ^fg^	n.d.	n.d.	n.d.	0.53 ± 0.6 ^fg^	0.53 ± 0.6 ^fg^	1.83 ± 0.09 ^r^
	**Family *Cyprinidae***						
*A. brama*	0.19 ± 0.02 ^a^	n.d.	n.d.	n.d.	0.19 ± 0.01 ^a^	0.19 ± 0.02 ^a^	n.d.
*A. mento*	0.54 ± 0.05 ^g^	n.d.	n.d.	n.d.	0.54 ± 0.05 ^gh^	0.54 ± 0.05 ^gh^	n.d.
*A. aspius*	0.41 ± 0.08 ^def^	n.d.	n.d.	n.d.	0.41 ± 0.08 ^def^	0.41 ± 0.08 ^def^	0.39 ± 0.02 ^jk^
*B. ballerus*	0.67 ± 0.4 ^hi^	0.04 ± 0.0 ^a^	n.d.	n.d.	0.71 ± 0.04 ^i^	0.67 ± 0.4 ^hi^	0.47 ± 0.02 ^l^
*B. bjoerkna*	1.68 ± 0.06 ^l^	n.d.	n.d.	n.d.	1.68 ± 0.06 ^l^	1.68 ± 0.06 ^l^	n.d.
*B. sapa*	0.18 ± 0.01 ^a^	n.d.	n.d.	n.d.	0.18 ± 0.01 ^a^	0.18 ± 0.01 ^a^	0.29 ± 0.03 ^gh^
*B. tauricus*	0.25 ± 0.04 ^bc^	0.18 ± 0.08 ^b^	n.d.	0.05 ± 0.02 ^a^	0.43 ± 0.08 ^de^	0.27 ± 0.05 ^bc^	0.96 ± 0.06 ^o^
*C. auratus*	0.55 ± 0.04 ^gh^	n.d.	n.d.	n.d.	0.55 ± 0.4 ^gh^	0.55 ± 0.04 ^gh^	0.19 ± 0.02 ^ef^
*C. carpio*	0.21 ± 0.02 ^b^	n.d.	n.d.	n.d.	0.21 ± 0.02 ^b^	0.21 ± 0.02 ^b^	n.d.
*H. molitrix*	0.36 ± 0.03 ^cd^	n.d.	n.d.	n.d.	0.36 ± 0.03 ^cd^	0.36 ± 0.03 ^cd^	0.28 ± 0.02 ^gh^
*P. cultratus*	0.25 ± 0.02 ^bc^	n.d.	n.d.	n.d.	0.25 ± 0.02 ^bc^	0.25 ± 0.02 ^bc^	0.18 ± 0.02 ^def^
*R. caspicus*	0.25 ± 0.02 ^bc^	n.d.	n.d.	n.d.	0.25 ± 0.02 ^bc^	0.25 ± 0.02 ^bc^	n.d.
*R. heckelii*	0.89 ± 0.05 ^j^	n.d.	n.d.	n.d.	0.89 ± 0.05 ^j^	0.89 ± 0.5 ^j^	0.31 ± 0.02 ^hi^
*S. erythrophthalmus*	2.77 ± 0.04 ^m^	n.d.	n.d.	0.06 ± 0.02 ^a^	2.83 ± 0.04 ^m^	2.78 ± 0.04 ^m^	0.24 ± 0.04 ^fg^
*V. vimba*	0.49 ± 0.08 ^efg^	n.d.	n.d.	n.d.	0.49 ± 0.08 ^efg^	0.49 ± 0.08 ^efg^	0.19 ± 0.01 ^ef^
	**Family *Gadidae***						
*G. chalcogrammus*	0.54 ± 0.01 ^g^	n.d.	n.d.	n.d.	0.54 ± 0.01 ^g^	0.54 ± 0.01 ^g^	n.d.
*G. morhua*	0.76 ± 0.08 ^i^	n.d.	n.d.	n.d.	0.76 ± 0.08 ^i^	0.76 ± 0.08 ^i^	0.34 ± 0.03 ^hij^
	**Family *Mullidae***						
*M. barbatus*	0.16 ± 0.02 ^a^	n.d.	n.d.	n.d	0.16 ± 0.02 ^a^	0.16 ± 0.02 ^a^	n.d.
	**Family *Osmeridae***						
*O. mordax*	0.16 ± 0.03 ^a^	n.d.	n.d.	n.d.	0.16 ± 0.03 ^a^	0.16 ± 0.03 ^a^	0.45 ± 0.05 ^kl^
*S. lucioperca*	0.19 ± 0.02 ^a^	n.d.	n.d.	n.d.	0.19 ± 0.02 ^a^	0.19 ± 0.02 ^a^	n.d.
	**Family *Percidae***						
*P. fluviatilis*	0.54 ± 0.02 ^g^	n.d.	n.d.	n.d.	0.54 ± 0.02 ^g^	0.54 ± 0.02 ^g^	0.13 ± 0.07 ^cde^
	**Family *Pleuronectidae***						
*P. quadrituberculatus*	3.14 ± 0.06 ^n^	0.04 ± 0.01 ^a^	n.d.	n.d.	3.18 ± 0.06 ^n^	3.15 ± 0.06 ^n^	0.54 ± 0.03 ^m^
**Family *Salmonidae***							
*O. gorbuscha*	0.17 ± 0.01 ^a^	n.d.	n.d.	n.d.	0.17 ± 0.0 ^a^	0.17 ± 0.01 ^a^	0.11 ± 0.11 ^bcd^
**Family *Siluridae***							
*P. asotus*	0.18 ± 0.01 ^a^	n.d.	n.d.	n.d.	0.18 ± 0.01 ^a^	0.18 ± 0.01 ^a^	0.58 ± 0.04 ^m^
**Roes**	**Family *Cyprinidae***						
*A. brama*	0.54 ± 0.19 ^p^	0.19 ± 0.0 ^b^	0.09 ± 0.2 ^a^	n.d.	0.56 ± 0.18 ^p^	5.45 ± 0.19 ^p^	0.54 ± 0.01 ^m^
*C. carpio*	3.08 ± 0.11 ^n^	n.d.	n.d.	n.d.	3.08 ± 0.1.1 ^n^	3.08 ± 0.11 ^n^	0.08 ± 0.01 ^bc^
*R. caspicus*	1.05 ± 0.09 ^k^	n.d.	n.d.	n.d.	1.05 ± 0.09 ^k^	1.05 ± 0.09 ^k^	0.70 ± 0.01 ^n^
*S. erythrophthalmus*	3.44 ± 0.08 ^o^	n.d.	n.d.	n.d.	3.44 ± 0.08 ^o^	3.44 ± 0.08 ^o^	0.05 ± 0.01 ^ab^
**Family *Moridae***							
*L. longipes*	0.89 ± 0.01 ^j^	n.d.	n.d.	n.d.	0.89 ± 0.01 ^a^	0.89 ± 0.01 ^j^	0.59 ± 0.02 ^m^
**Family *Osmeridae***							
*O. mordax*	0.90 ± 0.0 ^j^	n.d.	n.d.	n.d.	0.90 ± 0.0 ^a^	0.90 ± 0.01 ^j^	0.39 ± 0.00 ^jk^
	**Family *Pleuronectidae***						
*P. quadrituberculatus*	6.23 ± 0.09 ^q^	n.d.	0.08 ± 0.03 ^a^	n.d.	6.31 ± 0.09 ^q^	6.26 ± 0.0 ^q^	0.16 ± 0.09 ^de^

Within each column, different superscript letters indicate significant differences among values (*p* < 0.05) according to one-way ANOVA followed by Duncan’s test; n.d.: not detected (concentrations below LOQ, as reported in Supplementary File S1).

## Data Availability

Data is contained within the article or supplementary material.

## Supplementary Materials

The following supporting information can be downloaded at: https://www.mdpi.com/article/10.3390/foods12051083/s1, File S1: Supplementary File S1. Analytical Methodologies [12,14,35]; File S2: Supplementary Table S1. Fatty acid groups and ratios of dry-salted fish; File S3: Supplementary Table S2. Nutritional indices for fatty acids.

## Abbreviations

ANOVA: one-way analysis of variance; ALA: α-linolenic acid; ARA: arachidonic acid; CVD: cardiovascular disease; DHA: docosahexaenoic acid; DPA: docosapentaenoic acid; EPA: eicosapentaenoic acid; ETA: eicosatetraenoic acid; FA: fatty acid; FAME: FA methyl ester; FLQ: fish lipid quality; IA: index of atherogenicity; IT: index of thrombogenicity; HH: hypocholesterolemic/hypercholesterolemic ratio; HPLC: high-pressure liquid chromatography; LA: linoleic acid; MUFA: monounsaturated FA; OA: oleic acid; PA: palmitic acid; POA: palmitoleic acid; PUFA: polyunsaturated FA; SA: stearic acid; SDA: stearidonic acid; SFA: saturated FA; Sq: squalene; UFA: unsaturated FA; T3: tocotrienol; Tc: tocol; T: tocopherol; VA: vaccenic acid; VEA: vitamin E activity.

## References

[1] NowsadAlam A.K.M. (2007). Participatory Training of Trainers: A New Approach Applied in the Fish Processing.

[2] Mallick R., Basak S., Duttaroy A.K. (2021). Fatty acids and evolving roles of their proteins in neurological, cardiovascular disorders and cancers. Prog. Lipid Res..

[3] Huynh M.D., Kitts D.D. (2009). Evaluating nutritional quality of pacific fish species from fatty acid signatures. Food Chem..

[4] Djuricic I., Calder P.C. (2021). Beneficial outcomes of omega-6 and omega-3 polyunsaturated fatty acids on human health: An update for 2021. Nutrients.

[5] Nakamura M.T., Nara T.Y. (2003). Essential fatty acid synthesis and its regulation in mammals. Prostaglandins Leukot. Essent. Fat. Acids.

[6] EFSA (2017). Dietary Reference Values for nutrients Summary report. EFSA Support. Publ..

[7] Elagizi A., Lavie C.J., O’keefe E., Marshall K., O’keefe J.H., Milani R.V. (2021). An update on omega-3 polyunsaturated fatty acids and cardiovascular health. Nutrients.

[8] Delgado A., Al-Hamimi S., Ramadan M.F., Wit M.D., Durazzo A., Nyam K.L., Issaoui M. (2020). Contribution of tocols to food sensorial properties, stability, and overall quality. J. Food Qual..

[9] Traber M.G., Atkinson J. (2007). Vitamin E, antioxidant and nothing more. Free Radic. Biol. Med..

[10] Jiang Q. (2014). Natural forms of vitamin E: Metabolism, antioxidant, and anti-inflammatory activities and their role in disease prevention and therapy. Free Radic. Biol. Med..

[11] Ibrahim N.I., Naina Mohamed I. (2021). Interdependence of Anti-Inflammatory and Antioxidant Properties of Squalene–Implication for Cardiovascular Health. Life.

[12] Lyashenko S., González-Fernández M.J., Gomez-Mercado F., Yunusova S., Denisenko O., Guil-Guerrero J.L. (2019). Ribes taxa: A promising source of γ-linolenic acid-rich functional oils. Food Chem..

[13] Chen J., Liu H. (2020). Nutritional indices for assessing fatty acids: A mini-review. Int. J. Mol. Sci..

[14] Fabrikov D., Guil-Guerrero J.L., González-Fernández M.J., Rodríguez-García I., Gómez-Mercado F., Urrestarazu M., Lao M.T., Rincón-Cervera M.A., Álvaro J.E., Lyashenko S. (2019). Borage oil: Tocopherols, sterols and squalene in farmed and endemic-wild *Borago* species. J. Food Compos. Anal..

[15] FAO (2001). Human Vitamin and Mineral Requirements.

[16] USDA National Nutrient Database for Standard Reference. https://ndb.nal.usda.gov/ndb/.

[17] Citil O.B., Kalyoncu L., Kahraman O. (2014). Fatty acid composition of the muscle lipids of five fish species in Işıklı and Karacaören Dam Lake, Turkey. Vet. Med. Int..

[18] Guler G.O., Kiztanir B., Aktumsek A., Citil O.B., Ozparlak H. (2008). Determination of the seasonal changes on total fatty acid composition and ω3/ω6 ratios of carp (*Cyprinus carpio* L.) muscle lipids in Beysehir Lake (Turkey). Food Chem..

[19] Jobling M., Leknes O., Sæther B.S., Bendiksen E.Å. (2008). Lipid and fatty acid dynamics in Atlantic cod, *Gadus morhua*, tissues: Influence of dietary lipid concentrations and feed oil sources. Aquaculture.

[20] Ospina-E J.C., Sierra-C A., Ochoa O., Pérez-Álvarez J.A., Fernández-López J. (2012). Substitution of saturated fat in processed meat products: A review. Crit. Rev. Food Sci. Nutr..

[21] Wei Y., Meng Y., Li N., Wang Q., Chen L. (2021). The effects of low-ratio n-6/n-3 PUFA on biomarkers of inflammation: A systematic review and meta-analysis. Food Funct..

[22] Roy V.C., Park J.S., Ho T.C., Chun B.S. (2022). Lipid indices and quality evaluation of omega-3 rich oil from the waste of Japanese spanish mackerel extracted by supercritical CO_2_. Mar. Drugs.

[23] Rincón-Cervera M.A., González-Barriga V., Romero J., Rojas R., López-Arana S. (2020). Quantification and distribution of omega-3 fatty acids in South Pacific fish and shellfish species. Foods.

[24] Szewczyk K., Chojnacka A., Górnicka M. (2021). Tocopherols and tocotrienols—Bioactive dietary compounds; what is certain, what is doubt?. Int. J. Mol. Sci..

[25] Syväoja E.L., Salminen K., Piironen V., Varo P., Kerojoki O., Koivistoinen P. (1985). Tocopherols and tocotrienols in Finnish foods: Fish and fish products. J. Am. Oil Chem. Soc..

[26] Özogul F., Özogul Y., Kuley E. (2011). Simple extraction and rapid HPLC method for tocopherol analysis in marine and fresh-water fish species. Food Sci. Technol. Res..

[27] Franke A.A., Murphy S.P., Lacey R., Custer L.J. (2007). Tocopherol and tocotrienol levels of foods consumed in Hawaii. J. Agric. Food Chem..

[28] Kiczorowska B., Samolińska W., Grela E.R., Bik-Małodzińska M. (2019). Nutrient and mineral profile of chosen fresh and smoked fish. Nutrients.

[29] European Parliament, Council of the European Union (2006). Regulation (EC) No 1924/2006 of the European Parliament and of the Council of 20 December 2006 on nutrition and health claims made on foods. Off. J. Eur. Union.

[30] European Parliament, Council of the European Union (2011). Regulation (EU) No 1169/2011 of the European Parliament and of the Council of 25 October 2011 on the provision of food information to consumers, amending Regulations (EC) No 1924/2006 and (EC) No 1925/2006 of the European Parliament and of the Council, and repealing Commission Directive 87/250/EEC, Council Directive 90/496/EEC, Commission Directive 1999/10/EC, Directive 2000/13/EC of the European Parliament and of the Council, Commission Directives 2002/67/EC and 2008/5/EC and Commission Regulation (EC) No 608/2004. Off. J. Eur. Union.

[31] Kopicová Z., Vavreinová S. (2007). Occurrence of squalene and cholesterol in various species of Czech freshwater fish. Czech J. Food Sci..

[32] Ramírez-Torres A. (2011). Squalene: Current Knowledge and Potential Therapeutical Uses.

[33] Kloss L., Meyer J.D., Graeve L., Vetter W. (2015). Sodium intake and its reduction by food reformulation in the European Union—A review. NFS J..

[34] (2004). Standard for Salted Atlantic Herring and Salted Sprat.

[35] AOAC (1995). Association of Official Analytical Chemists, Methods of Analysis.

