# Supplementary material for: Screening of Lesser-Known Salted–Dried Fish Species for Fatty Acids, Tocols, and Squalene
Source: Foods. 2023 Mar 3;12(5):1083. doi: 10.3390/foods12051083 (PMC10000464; doi:10.3390/foods12051083)
Supplement: Supplementary file 1 [file foods-12-01083-s001.zip › Supplementary File S1. Analytical Methodologies.pdf]

Manuscript: *Screening of Lesser-Known Salted–Dried Fish Species for Fatty Acids, Tocols, and Squalene*

Supplementary File S1. Material and Methods

1. Moisture content

Moisture content of dried fish and roes was analysed in triplicate according to the standard procedure given by the Association of Official Analytical Chemists (AOAC 1995).

2. Fatty acids analyses

FA profiles were obtained after direct derivatization of samples to yield FA methyl esters (FAME). For this, 250 mg of each sample were placed in test tubes and then 50 µL of the internal standard (heptadecanoic acid, 17:0, Sigma-H3500; Sigma-Aldrich, Barcelona, Spain) in *n*-hexane (10 mg/mL) were added. After that, 2 mL of a methylating mixture (methanol:acetyl chloride, 20:1, v/v) and 1 mL *n*-hexane were carefully poured over the described material. Tubes were then capped and heated at 100 °C for 30 min. After tubes were cooled to room temperature, 1 mL of distilled water was added to each tube and then centrifuged for 5 min at 2000g on a Heraeus Labofuge 200 centrifuge (Thermo Scientific, USA). Finally, the hexane layer was collected for FAME analysis by gas chromatography (GC) using a Focus GC (Thermo Electron, Cambridge, UK), equipped with a flame ionization detector (FID) and an Omegawax 250 capillary column (30 m × 0.25 mm i.d. × 0.25 µm film thickness; Supelco. Bellefonte, USA), as previously described (Lyashenko et al., 2019). The peak area of the internal standard was used as reference to calculate the mass of each FA in the resulting chromatograms to report the total amount of FA per 100 g sample, and FA profiles were shown as FA percentages of total FA (Table 2). Peaks were identified by retention times obtained for known FAME standards (PUFA No. 1, 47033; methyl γ-linolenate ≥99% purity, L6503; and methyl stearidonate 97% purity, 43959 FLUKA) from Sigma-Aldrich (Barcelona, Spain). The LOD was defined as the minimum concentration at which distinct peaks were detected above the baseline noise. The LOQ was defined as the lowest concentration of FA that could be quantified with an accuracy and precision within 15%. The estimated LODs for EPA and DHA were in the 0.4-0.5 µg/mLrange, while LOQs for such molecules were in the 2.0–3.1 µg/mL range. The recovery was calculated by the formula: [C(Exp)/C(Theo) x 100].

Recoveries for EPA were in the 95.8–100% range, those for DHA were in the 99.3–100% range. As a GC quality control, a blank sample (hexane) was run together with the samples in every batch. Control oil samples (Canola oil, 46961, from Sigma-Aldrich) were analyzed before and after running samples.

3. Extraction of tocols and squalene

This procedure was effected as described in a previous paper of our research group (Fabrikov et al., 2019). Approximately 0.2-0.3 g of sample (either fish fillets or roes) was weighed in a 100 mL screw-cap flask. Solutions of ascorbic acid (0.1 M, 5 mL) and potassium hydroxide (2 M, 20 mL) were added for further heating (45 min at 60 °C). To extract the unsaponifiable components, the mixture was filtered and then 10 mL of saturated NaCl solution and 10 mL of *n*-hexane with BHT (5 mg/L) were added. After vortexing for 1 min, the organic layer was collected in a 50 mL round bottom flask. The aqueous layer was extracted again with 5 mL of *n*-hexane and then both hexane layers were combined. The *n*-hexane was removed in a rotary evaporator (Heidolph, Hei-Vap, Germany) at 60 °C until dryness and the resulting residue was dissolved in 1 mL of 2-propanol. The extracts were stored at -20 ºC in darkness under an inert atmosphere of nitrogen until HPLC analysis.

4. Tocopherol and tocotrienol analyses

Both tocopherols (T) and tocotrienols (T_3) were identified by chromatographic comparison with pure standards (Sigma-Aldrich, Barcelona, Spain), by co-elution and by their UV spectral characteristics (DAD). Peaks purity evaluation on the DAD measurements was based on spectral comparison at three different peak heights. T and T_3 homologs were determined using RP-HPLC/DAD (Agilent 1100 series, Palo Alto, CA, USA) equipped with a ProntoSIL C30 column (4.6 × 250 mm, 3 μm; Bischoff Chromatography, Leonberg, Germany) cooled at 15 °C. Mixtures of methanol:acetonitrile (95:5, v/v, phase A) and 2-propanol:*n*-hexane (50:50, v/v, phase B) were used as mobile phase at a flow 7 rate of 0.8 mL/min. The following sequence was used to elute each sample: 25 min of phase A (100 %) followed by 20 min of phase B (100 %). Additional 15 min of phase A (100%) were used to re-equilibrate the column. Phase B was used as a washing solution. The wavelength selected for DAD was 290 nm. Limit of detection (LOD), limit of quantification (LOQ), linear range and recoveries rates were obtained as previously reported (Fabrikov et al., 2019). For α-T, LOD and LOQ were 0.40 and 0.90 mg/L; linear range – (R^2) was 0.90−200 mg/L (0.9989); and the recovery rate was 101.2±1.1%. For β-T, LOD and LOQ were 0.25 and 0.60 mg/L; linear

range – (R^2) was 0.60−250 mg/L (0.9991); and the recovery rate was 100.4±2.3%. For γ-T, LOD and LOQ were 0.15 and 0.40 mg/L; linear range – (R^2) was 0.4−200 mg/L (0.9992); and the recovery rate was 98.3±0.9%. For δ-T, LOD and LOQ were 0.25 and 1.20 mg/L; linear range – (R^2) was 1.20−200 mg/L (0.9990); and the recovery rate was 101.8±1.7. %. For α-T_3, LOD and LOQ were 0.19 and 0.46 mg/L; linear range – (R^2) was 0.46−200 mg/L (0.9997); and the recovery rate was 98.6±1.3%. For β-T_3, LOD and LOQ were 0.16 and 0.30 mg/L; linear range – (R^2) was 0.3−250 mg/L (0.9989); and the recovery rate was 101.6±1.8%. For γ-T_3, LOD and LOQ were 0.12 and 0.25 mg/L; linear range – (R^2) was 0.20−200 mg/L (0.9998); and the recovery rate was 99.2±1.7%. For δ-T_3, LOD and LOQ were 0.25 and 1.20 mg/L; linear range – (R^2) was 0.25−200 mg/L (0.9993); and the recovery rate was 100.3±1.1%.

5. Squalene analyses

The protocol for the extraction of squalene was the same to that developed for T and T_3, and it was determined by RP-HPLC/DAD according to Fabrikov et al. (2019). In this case, a Luna C18 column (250×4.6 mm, 5 µm; Phenomenex) was used at 30 °C. The mobile phase was a mixture of methanol:acetonitrile 70:30 (v/v), and it was programmed in isocratic mode with a flow rate of 0.8 mL/min for 55 min. The results were expressed in µg/g of sample.

Squalene was measured at 210 nm and quantified by means of a calibration curve made with a squalene standard (≥98%) from Sigma-Aldrich (Barcelona, Spain). LOD and LOQ were 0.15 and 0.50 mg/L, respectively; and the linearity range – (R^2) was 0.50 − 90 mg/L (0.9899). This methodology was previously validated in our research groups through checking linearity, sensitivity and accuracy (Fabrikov et al., 2019).

References

AOAC (1995). Association of Official Analytical Chemists. Official methods of analysis. 16 ed. Virginia, USA: Association of Official Analytical Chemists.

Fabrikov, D., Guil-Guerrero, J. L., González-Fernández, M. J., Rodríguez-García, I., Gómez-Mercado, F., Urrestarazu, M., Lao, M. T., Rincón-Cervera, M. A., Álvaro, J. E., Lyashenko, S. (2019). Borage oil: Tocopherols, sterols and squalene in farmed and endemic-wild Borago species. *Journal of Food Composition and Analysis*, *83*, 103299.

Lyashenko, S., González-Fernández, M. J., Gomez-Mercado, F., Yunusova, S., Denisenko, O., Guil-Guerrero, J. L. (2019). *Ribes* taxa: A promising source of γ-linolenic acid-rich functional oils. *Food chemistry*, *301*, 125309.
