# Supplementary material for: Screening of Lesser-Known Salted–Dried Fish Species for Fatty Acids, Tocols, and Squalene
Source: Foods. 2023 Mar 3;12(5):1083. doi: 10.3390/foods12051083 (PMC10000464; doi:10.3390/foods12051083)
Supplement: Supplementary file 1 [file foods-12-01083-s001.zip › Supplementary Table S1. Fatty acids groups and ratios of dry-salted fish.pdf]

Manuscript: *Screening of Lesser-Known Salted–Dried Fish Species for Fatty Acids, Tocols, and Squalene*

Supplementary Table S1. Fatty acids groups and ratios of salted-dried fish

	∑ SFA	∑ MUFA	∑ PUFA	∑ *n*-6 PUFA	∑ *n*-3 PUFA	∑ EPA+DHA	DHA/EPA
**Fillets**				**Family *Clupeidae***			
A. kessleri	24.4	49.0	19.6	3.0	16.6	12.7	1.35
C. cultriventris	31.3	49.3	19.4	1.8	17.6	12.4	2.54
				**Family *Cyprinidae***			
A. brama	26.2	33.9	35.7	6.1	29.6	24.5	1.69
A. mento	31.3	31.9	31.0	3.5	27.5	16.8	1.63
A. aspius	36.9	40.6	13.1	2.7	10.4	8.8	4.18
B. ballerus	32.1	23.5	41.8	7.8	34.0	28.7	2.26
B. sapa	35.8	21.9	41.5	10.4	31.1	26.2	2.01
B. tauricus	21.1	31.1	37.1	22.4	14.7	8.9	2.18
B. bjoerkna	32.9	37.5	22.3	4.1	18.2	13.6	1.34
C. auratus	30.2	22.4	38.2	9.4	28.8	25.0	2.68
C. carpio	35.7	23.7	33.0	5.9	27.1	19.4	1.52
H. molitrix	39.6	38.7	23.0	6.3	16.7	12.5	1.40
P. cultratus	38.2	42.8	15.8	3.7	12.1	9.8	3.67
R. caspicus	28.5	29.9	37.1	4.6	32.5	28.8	1.97
R. heckelii	31.0	45.1	23.8	4.9	18.9	14.9	1.10
S. erythrophthalmus	33.3	24.9	37.8	16.0	21.8	15.0	2.57
V. vimba	28.9	32.1	34.5	7.7	26.8	19.0	2.06
				**Family *Gadidae***			
G. chalcogrammus	31.6	20.2	48.1	4.2	43.9	39.8	3.80
G. morhua	20.6	33.0	44.3	2.1	42.2	41.0	1.29
				**Family *Percidae***			
P. fluviatilis	33.3	12.6	47.3	4.8	42.5	34.4	8.05
				**Family *Osmeridae***			
O. mordax	31.2	28.4	39.1	1.7	37.4	34.7	1.84
S. lucioperca	29.3	26.1	40.8	3.9	36.9	32.9	5.45
				**Family *Carangidae***			
S. leptolepis	38.7	15.1	43.0	5.7	37.3	31.3	4.22
S. quinqueradiata	35.5	12.3	49.7	3.2	46.5	39.4	6.88
				**Family *Mullidae***			
M. barbatus	35.4	29.6	33.6	6.3	27.3	23.2	5.82
				**Family *Pleuronectidae***			
P. quadrituberculatus	20.9	37.0	35.9	3.0	32.9	27.5	0.29
				**Family *Salmonidae***			
O. gorbuscha	30.5	22.3	32.1	1.9	30.2	22.4	1.95
				**Family *Siluridae***			
P. asotus	32.2	37.2	25.9	7.5	18.4	11.4	1.59
**Roes**				**Family Cyprinidae**			
A. brama	34.0	27.2	36.8	5.1	31.7	26.9	2.79
C. carpio	29.6	29.2	30.5	7.5	23.0	17.1	3.07
R. caspicus	32.2	21.2	44.8	4.0	40.8	35.7	1.73
S. erythrophthalmus	33.3	24.9	37.8	16.0	21.8	15.0	2.57
				**Family Moridae**			
L. longipes	34.9	33.5	29.4	6.5	22.9	16.6	3.05
				**Family *Osmeridae***			
O. mordax	27.9	36.9	32.7	2.0	30.7	26.9	0.64
				**Family *Pleuronectidae***			
P. quadrituberculatus	26.4	15.8	46.0	3.0	43.0	37.3	0.98
