# Supplementary material for: Screening of Lesser-Known Salted–Dried Fish Species for Fatty Acids, Tocols, and Squalene
Source: Foods. 2023 Mar 3;12(5):1083. doi: 10.3390/foods12051083 (PMC10000464; doi:10.3390/foods12051083)
Supplement: Supplementary file 1 [file foods-12-01083-s001.zip › Supplementary Table S2. Fish nutritional indices.pdf]

# Manuscript: *Screening of Lesser-Known Salted–Dried Fish Species for Fatty Acids, Tocols, and Squalene*

## Supplementary Table S2. Nutritional Indices for Fatty Acids

| Species | PUFA/SFA | *n*-6/*n*-3 PUFA ratio | EPA+DHA | AI | TI | HH | FLQ |
|---|---|---|---|---|---|---|---|
| **Fillets** | | | | **Family *Carangidae*** | | | |
| *S. leptolepis* | 1.11 | 0.15 | 31.3 | 0.48 | 0.29 | 2.28 | 32.33 |
| *S. quinqueradiata* | 1.40 | 0.07 | 39.4 | 0.36 | 0.21 | 3.12 | 40.41 |
| | | | | **Family: *Clupeidae*** | | | |
| *A. kessleri* | 0.80 | 0.18 | 12.7 | 0.39 | 0.28 | 2.82 | 13.42 |
| *C. cultriventris* | 0.62 | 0.10 | 12.4 | 0.57 | 0.31 | 2.27 | 12.46 |
| | | | | **Family *Cyprinidae*** | | | |
| *A. brama* | 1.36 | 0.21 | 24.5 | 0.35 | 0.20 | 3.46 | 25.31 |
| *A. mento* | 0.99 | 0.13 | 16.8 | 0.61 | 0.27 | 2.06 | 17.97 |
| *A. aspius* | 0.36 | 0.26 | 8.8 | 0.62 | 0.56 | 1.80 | 9.70 |
| *B. ballerus* | 1.30 | 0.23 | 28.7 | 0.45 | 0.26 | 2.41 | 29.47 |
| *B. bjoerkna* | 0.68 | 0.23 | 13.6 | 0.55 | 0.39 | 2.28 | 14.36 |
| *B. sapa* | 1.16 | 0.33 | 26.2 | 0.45 | 0.31 | 2.31 | 26.41 |
| *B. tauricus* | 1.76 | 1.52 | 8.9 | 0.24 | 0.23 | 4.85 | 9.92 |
| *C. auratus* | 1.26 | 0.33 | 25.0 | 0.43 | 0.28 | 2.29 | 27.90 |
| *C. carpio* | 0.92 | 0.22 | 19.4 | 0.35 | 0.30 | 3.03 | 20.86 |
| *H. molitrix* | 0.58 | 0.38 | 12.5 | 0.73 | 0.46 | 1.91 | 12.72 |
| *P. cultratus* | 0.41 | 0.31 | 9.8 | 0.53 | 0.57 | 2.00 | 10.01 |
| *R. caspicus* | 1.30 | 0.14 | 28.8 | 0.41 | 0.23 | 2.65 | 29.72 |
| *R. heckelii* | 0.77 | 0.26 | 14.9 | 0.45 | 0.34 | 2.27 | 15.11 |
| *S. erythrophthalmus* | 1.14 | 0.73 | 15.0 | 0.46 | 0.32 | 2.33 | 15.63 |
| *V. vimba* | 1.19 | 0.29 | 19.0 | 0.39 | 0.26 | 2.73 | 19.77 |
| | | | | **Family *Gadidae*** | | | |
| *G. chalcogrammus* | 1.52 | 0.10 | 39.8 | 0.44 | 0.20 | 2.35 | 39.84 |
| *G. morhua* | 2.15 | 0.05 | 41.0 | 0.31 | 0.13 | 3.76 | 41.88 |
| | | | | **Family *Mullidae*** | | | |
| *M. barbatus* | 0.95 | 0.23 | 23.2 | 0.47 | 0.32 | 2.80 | 23.53 |
| | | | | **Family *Osmeridae*** | | | |
| *O. mordax* | 1.25 | 0.05 | 34.7 | 0.55 | 0.20 | 2.35 | 34.91 |
| *S. lucioperca* | 1.39 | 0.11 | 32.9 | 0.39 | 0.21 | 2.76 | 34.16 |
| | | | | **Family *Percidae*** | | | |
| *P. fluviatilis* | 1.42 | 0.11 | 34.4 | 0.39 | 0.20 | 2.83 | 37.55 |
| | | | | **Family *Pleuronectidae*** | | | |
| *P. quadrituberculatus* | 1.72 | 0.09 | 27.5 | 0.40 | 0.15 | 3.20 | 29.13 |
| | | | | **Family *Salmonidae*** | | | |
| *O. gorbuscha* | 1.05 | 0.06 | 22.4 | 0.52 | 0.17 | 3.09 | 26.38 |
| | | | | **Family *Siluridae*** | | | |
| *P. asotus* | 0.80 | 0.41 | 11.4 | 0.42 | 0.37 | 2.54 | 11.89 |
| **Roes** | | | | **Family *Cyprinidae*** | | | |
| *A. brama* | 1.08 | 0.16 | 26.9 | 0.46 | 0.25 | 2.34 | 27.56 |
| *C. carpio* | 1.03 | 0.33 | 17.1 | 0.41 | 0.30 | 2.39 | 19.48 |
| *R. caspicus* | 1.39 | 0.10 | 35.7 | 0.42 | 0.21 | 2.45 | 36.35 |
| *S. erythrophthalmus* | 1.12 | 0.28 | | 0.47 | 0.31 | 1.99 | 26.85 |
| | | | | **Family *Moridae*** | | | |
| *L. longipes* | 0.84 | 0.28 | 16.6 | 0.45 | 0.37 | 2.18 | 16.97 |
| | | | | **Family *Osmeridae*** | | | |
| *O. mordax* | 1.17 | 0.07 | 26.9 | 0.61 | 0.21 | 2.22 | 27.59 |
| | | | | **Family *Pleuronectidae*** | | | |
| *P. quadrituberculatus* | 1.74 | 0.07 | 37.3 | 0.43 | 0.16 | 2.84 | 42.10 |

**Abbreviations:** PUFA: polyunsaturated fatty acids: SFA: saturated fatty acids, AI: atherogenic index; TI: thrombogenic index; HH: hypocholesterolemic/hypercholesterolemic fatty acids ratio; FLQ: fish lipid quality.
